# Development of a qualitative data analysis codebook informed by the i-PARIHS framework

**DOI:** 10.1186/s43058-022-00344-9

**Published:** 2022-09-14

**Authors:** Mona J. Ritchie, Karen L. Drummond, Brandy N. Smith, Jennifer L. Sullivan, Sara J. Landes

**Affiliations:** 1 Department of Veterans Affairs, Behavioral Health Quality Enhancement Research Initiative (QUERI), 2200 Ft Roots Dr, North Little Rock, AR 72114 USA; 2 Department of Psychiatry, University of Arkansas for Medical Sciences, 4301 W Markham St., #755, Little Rock, AR 72205 USA; 3 Center for Innovation in Long Term Services and Supports, VA Providence Healthcare System, 385 Niagara St, Providence, RI 02907 USA; 4 Department of Health Service, Policy and Practice, School of Public Health, Brown University, 121 S Main St, Providence, RI 02906 USA

**Keywords:** Integrated-Promoting Action on Research Implementation in Health Services framework, i-PARIHS, Qualitative methods, Implementation science

## Abstract

**Background:**

The Integrated-Promoting Action on Research Implementation in Health Services (i-PARIHS) framework and its predecessor, PARIHS, have been widely utilized in implementation studies. Although i-PARIHS developers have focused on creating tools to guide facilitators in its application in practice, tools are also needed for evaluation and research. Codebooks with clear and meaningful code labels and definitions are an important component of qualitative data analysis and have been developed for other widely used frameworks. There is no such codebook for i-PARIHS. Additionally, sub-constructs for the Innovation, Recipients, and Context constructs lack definitions, and there is no sub-classification of facilitation activities for the Facilitation construct. The lack of a standardized codebook hinders our ability to synthesize research findings across studies, explore and test the range of activities that are utilized in facilitation efforts, and potentially validate and further refine i-PARIHS. This paper describes a rigorous process of developing a detailed qualitative codebook informed by the i-PARIHS framework.

**Methods:**

A workgroup of qualitative researchers conducted a rigorous four-phase process to develop a codebook informed by i-PARIHS. In phase 1, workgroup members reviewed and discussed literature, consulted an organizational scientist, and drafted and refined subcodes and definitions for i-PARIHS constructs. In phase 2, they obtained feedback from an expert panel and further refined subcodes and definitions. In phase 3, they obtained feedback from i-PARIHS developers/experts and incorporated it into the codebook. Finally, two studies piloted the application of the codebook which informed the final version.

**Results:**

The resulting i-PARIHS-informed codebook includes definitions for the four main constructs of the framework: Innovation, Recipients, Context, and Facilitation; subcodes and definitions for characteristics of each of these constructs; and instructions for the suggested application of individual codes and use of the codebook generally.

**Conclusions:**

The standardized codes and definitions in the codebook can facilitate data exploration, pattern identification, and insight development informed by the i-PARIHS framework. Qualitative analysts can also use them to explore interactions between i-PARIHS constructs, maximize the potential for comparing findings across studies, and support the refinement of the i-PARIHS framework using empirical findings from multiple studies.

**Supplementary Information:**

The online version contains supplementary material available at 10.1186/s43058-022-00344-9.

Contributions to the literature

- The i-PARIHS framework is widely utilized in implementation studies to inform data analysis, but it does not include well-defined sub-constructs that can be used to code qualitative material.
- This paper presents a qualitative codebook informed by i-PARIHS and describes the rigorous process, including a review by i-PARIHS developers, used to create the codebook.
- The paper demonstrates how this codebook can be used to explore interactions between i-PARIHS constructs, including how facilitation activities address characteristics of the Innovation, Recipients, and Context.
- The use of the i-PARIHS-informed codebook will allow for the comparison of findings across studies, thus advancing our understanding of implementation processes, and support further refinements to the framework.

## Background

Scholars posit that conceptual approaches, i.e., theories, models, and/or frameworks (TMFs), should inform the implementation and evaluation of evidence-based practices and programs [1–4]. The Integrated-Promoting Action on Research Implementation in Health Services (i-PARIHS) framework is one of the more commonly used implementation frameworks [5, 6]. Its predecessor, the PARIHS framework, was specifically designed for implementing complex multi-disciplinary team-based innovations in healthcare settings and was one of the first frameworks to account for the complex nature of the implementation process [7, 8]. It had a rich history of refinement [9–13] ultimately leading to the development of the i-PARIHS framework [7]. Both PARIHS and i-PARIHS have been widely utilized to guide the assessment of factors that influence implementation, inform data collection and analysis, and evaluate study findings [5, 14, 15]. More recently, i-PARIHS has been increasingly utilized to prospectively plan what facilitators will do to support implementation [16], to inform what they are doing during the implementation process [17, 18], and to develop resources to train and support facilitators [17].

Two of the main strengths of the i-PARIHS framework are (1) its emphasis on the interaction between implementation influences and (2) the assertion that facilitation is the active ingredient for successfully implementing an innovation. The framework proposes SI = Fac (I + R + C), i.e., successful implementation (SI) is the result of the facilitation (Fac) of the Innovation (I) with the Recipients (R) in their context (C) [7, 19]. Characteristics of three of these constructs, the Innovation (the practice or program being implemented), the Recipients (the individuals and teams that influence and are affected by innovation implementation), and the Context (the setting within which implementation occurs), were identified by framework developers based on the theoretical and empirical literature, as well as their own experiences, and are typically considered to be implementation determinants [20]. Recognizing the complexity of implementation processes, i-PARIHS developers propose that the characteristics of these three constructs interact [15]. Facilitation, the fourth construct and a unique feature of i-PARIHS, is the active ingredient in implementation efforts, interacting with the characteristics of the other three constructs to maximize the potential for implementation success. Facilitation has been defined as a “multi-faceted interactive process of problem solving, enabling and supporting individuals, groups, and organizations in their efforts to adopt and incorporate innovations into routine practices” [21]. It is both a role (i.e., the facilitator) and a process (i.e., the activities that support and enable implementation) [7, 19]. Although the role of facilitator can be filled by individuals who are internal to systems implementing change, it can also be filled by experts who are external to such systems.

Despite the importance and value of applying TMFs, i.e., i-PARIHS, they are not always utilized or well-described in implementation studies [5, 22]. To foster their use, scholars have created tools to operationalize them [23, 24]. Developers of i-PARIHS have focused on creating tools for its application in practice by providing guidance to facilitators for fostering successful implementation [7, 17]. To facilitate the use of other TMFs in evaluation and research, scholars have developed measures for assessing constructs [25, 26], including qualitative interview guides and codebooks. Codebooks are an important component of the qualitative data analysis process; they facilitate data exploration, pattern identification, and insight development [27]. The use of a deductive codebook based on a TMF or a hybrid inductive-deductive codebook that includes the former as well as additional inductive codes can help focus the analysis of qualitative data, as well as test and refine the TMF. Standard operational definitions of constructs and sub-constructs have been developed for several TMFs, including the publicly available codebook created for the Consolidated Framework for Implementation Research (CFIR) [28, 29], a coding manual developed for the Normalization Process Theory [30], and a list of operationalized definitions for the Exploration, Preparation, Implementation, Sustainment (EPIS) framework [31, 32]. However, there is no such codebook or list of operational definitions for the i-PARIHS framework. Thus, individual studies develop their own codebooks, making it difficult to compare findings across studies using i-PARIHS.

There are a number of challenges to creating a qualitative codebook informed by i-PARIHS. Codebooks typically include code labels (usually a word or short phrase), definitions of labels, instructions for using them, and examples of representative data, The i-PARIHS framework was originally presented within the context of a guide for facilitation practitioners in which developers described, based on their own experiences, what facilitators should do to address characteristics of the innovation, recipients, and context [7]. Although i-PARIHS developers specify characteristics of the Innovation, Recipients, and Context constructs, these lack the formal definitions [5, 14] needed for a codebook. Additionally, the Facilitation construct is under-defined and there is no sub-classification of facilitation activities [14]. Consistent with its presentation within the context of a facilitation guide, i-PARIHS does include recommendations for facilitators, but many of the recommended actions are narrowly focused on one of the specific constructs and/or are very complex. No studies to date have used these recommendations as subcodes. The lack of clearly defined construct characteristics and sub-classification of facilitation activities hinders efforts to compare findings across studies, explore and test the range of activities that are utilized in facilitation efforts, and potentially validate or further refine i-PARIHS. This paper describes a rigorous four-phase process of developing a detailed qualitative codebook informed by i-PARIHS, including a sub-classification of facilitation activities, identified in previous work [33, 34], which can be used to explore how the characteristics of i-PARIHS constructs influence implementation and how they interact with each other in the process.

Established in 1998, the US Department of Veterans Affairs (VA) Quality Enhancement Research Initiative (QUERI) [35] currently funds more than 40 programs and centers across the US that leverage innovative scientific methods to accelerate evidence into practice and improve the quality and safety of care delivered to veterans. One of the first funded programs, now called Behavioral Health (BH) QUERI, was focused on improving behavioral health care. In 2016, BH QUERI began multiple implementation efforts utilizing the i-PARIHS framework to guide their implementation facilitation strategies, as well as data collection and analysis pertaining to factors determining implementation success. This work represented a unique opportunity to use common measures and methods to examine the effectiveness of facilitation across interventions, sites, and varying levels of facilitation. In 2017, i-PARIHS developers Gill Harvey and Alison Kitson invited BH QUERI to form a collaboration for purposes of knowledge sharing and advancing the study of facilitation. An early decision of this collaboration was that a common or standard codebook informed by the i-PARIHS framework would be helpful as a first step toward more robust comparisons of results across disparate projects using the framework. The BH QUERI team took responsibility for this work. Below we describe the process of codebook development.

## Methods

**Fig. 1:**
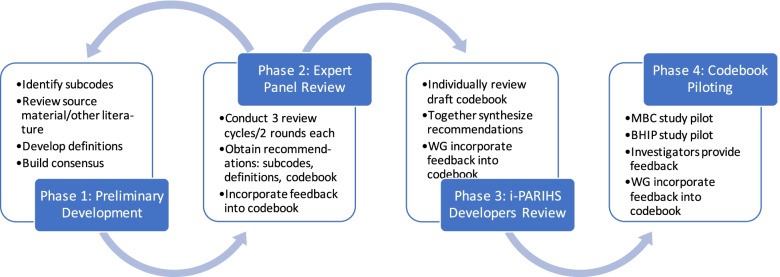
Summary of the codebook development process

All authors were members of a workgroup that planned and conducted a rigorous four-phase process to develop the codebook. MJR, KD, SL, and JS have expertise in implementation science (including implementation facilitation and the i-PARIHS framework) and qualitative methods. BS is a research assistant who had previous experience in using the original PARIHS framework in qualitative data analysis. Accepting the four i-PARIHS constructs as top-level codes, in phase 1, we identified preliminary subcodes for each of the constructs and developed subcode definitions and instructions for their use. In phase 2, we conducted an iterative expert panel process to obtain feedback from other implementation scientists who had expertise in qualitative methodology and i-PARIHS. In phase 3, we sought feedback from i-PARIHS developers/experts, and in phase 4, we piloted the application of the codebook in two studies (see Fig. 1 for a summary of the tasks within each phase). Below, we describe these phases in detail.

### Phase 1: Preliminary codebook development (September 2017 to November 2018)

#### Innovation, Recipients, and Context subcodes

For the Innovation, Recipients, and Context constructs, workgroup members assessed the names of i-PARIHS construct characteristics [7] for use as subcode labels. We adopted names we considered to be conceptually meaningful and clear and modified names or made recommendations for labels to maximize clarity. We assessed, based on literature and our experience, whether there were important characteristics of i-PARIHS constructs that were missing from source materials but should potentially be included as subcodes. Because there was significant overlap between the characteristics of the Recipient and Context constructs, we sought consultation from an organizational science and qualitative methodology expert to help us identify context subcodes and differentiate between Context and Recipient subcodes.

One or two workgroup members were responsible for drafting the initial definition and coding instructions for each subcode based on descriptions of the construct characteristic in core materials [7, 19, 36], literature related to theories that informed i-PARIHS developers’ understanding of the characteristic (e.g., Complexity Theory [7];), and other literature related to i-PARIHS or its predecessor, PARIHS (e.g., [37–39]). When they were still unable to create a clear definition, they reviewed other relevant literature (e.g., [28]) (see Additional file 1 for a list of literature reviewed in the development process). All other workgroup members individually reviewed the drafted material and provided feedback. The member responsible incorporated feedback and, if needed, reviewed additional literature and modified the definition and/or instructions. The workgroup members then reviewed and discussed the second iteration and suggested additional edits, if appropriate. The workgroup leader, MJR, incorporated these suggestions, as well as the consultant’s suggestions for context subcode definitions and coding instructions, and finalized the preliminary definition and instructions.

#### Facilitation Activity subcodes

Because i-PARIHS developers assert that facilitators activate implementation by assessing and responding to the Innovation, Recipients, and Context [7], we chose to focus subcodes for the Facilitation construct on activities that facilitators perform. Since the i-PARIHS Facilitation construct is under-defined and lacks sub-classification [14], we opted to use facilitation activities we had identified and described in previous work as the foundation for subcodes. In a large VA-funded project that applied and evaluated an implementation facilitation strategy, researchers had performed a targeted literature review, including empirical and conceptual literature about the PARIHS framework. They then conducted a directed content analysis of 85 qualitative interviews with three facilitators over a two- and one-half-year period to create a list of facilitation activities [33]. Building on this work, in preparation for another study, BH QUERI researchers conducted a comprehensive scoping review of facilitation literature from 1996 to 2015 and further refined this list, resulting in 32 facilitation activities with operational descriptions [34]. The workgroup reviewed and adopted this list of activities as the subcodes for the construct and further refined the previously developed descriptions of facilitation activities. Because of the large number of specific facilitation activity subcodes, we also clustered these subcodes to create higher-level codes to enable a more expedited analysis with the option to pursue more detailed sub-coding within specific clusters.

### Phase 2: Expert panel review (September 2018 to February 2019)

We recruited eight researchers who had expertise in implementation science (average of 7.8 years) and qualitative methodology (average of 12.6 years) to serve on an expert panel. Panel members rated their knowledge of the i-PARIHS framework on a scale from 1 to 5 (poor to excellent) and reported a median rating of 4 (very good). We conducted the expert panel review process in three cycles. In cycle 1, the panel reviewed and provided feedback on subcodes for the Innovation construct. In cycle 2, they reviewed and provided feedback on Facilitation Activity subcodes, and in cycle 3, on the Context and Recipients subcodes. Each cycle was completed in two rounds.

In round 1 of each cycle, we provided the panel with a document containing (a) information about the development process; (b) codebook material including construct subcodes, definitions, and coding instructions; (c) workgroup recommendations for combining, modifying, and excluding subcodes; and (d) questions for additional feedback. The document also provided instructions for the review process, asking panel members to (1) evaluate codebook material for clarity and comprehensiveness; (2) evaluate workgroup recommendations for changes and/or additions to i-PARIHS construct characteristics; and (3) provide written feedback, including recommendations for edits to codebook material. MJR reviewed round 1 feedback and contacted panel members individually, as needed, to clarify their recommendations. MJR and KLD then determined what material could be revised and finalized based upon round 1 results vs. material that would require further panel discussion in round 2; they updated the codebook accordingly.

In round 2 of each cycle, we provided the panel with a document containing (a) codebook material that we had been unable to finalize; (b) panel members’ recommendations from round 1; (c) if appropriate, additional material from the literature; and (d) instructions for this round of the review process. Panel members individually reviewed this material and then met together by phone to discuss and provide verbal feedback to MJR. MJR and KLD reviewed and came to a consensus on feedback that merited further discussion and incorporated this feedback to finalize the preliminary codebook.

### Phase 3: i-PARIHS developers review (February to July 2019)

To further refine the codebook, we obtained feedback from i-PARIHS framework developers, Drs. Gill Harvey and Alison Kitson and Dr. Sarah Hunter, a post-doctoral research fellow who was leading a team to develop tools and resources to support the use of the framework in research and clinical practice. We provided these experts with a draft of the codebook and workgroup lead MJR met with them by phone to orient them to the codebook, describe the workgroup’s development process, and ask them to review and provide feedback on the codebook as a whole and the subcodes, definitions, and coding instructions. The experts individually reviewed the codebook and then met face-to-face to discuss and synthesize their comments and recommendations into a single document, which they provided to the workgroup. MJR and KLD reviewed their comments and came to a consensus on changes to the codebook.

### Phase 4: Codebook piloting (July 2019 to April 2021)

To ensure that code labels, definitions, and instructions were clear, we piloted the application of the codebook in data collected for two BH QUERI projects, both informed by the i-PARIHS framework. In one project, facilitators had supported the implementation of measurement-based care (MBC) in VA primary care clinics [40]. In the other project, facilitators had supported the implementation of the collaborative care model in VA-based outpatient mental health teams, called Behavioral Health Interdisciplinary Program (BHIP) teams [41]. The results of these studies and a complete description of their methods will be presented in future publications.

MBC project researchers piloted the codebook on qualitative data collected for multiple study components. In total, the pilot included the application of all Innovation and Recipients codes and clustered Context and Facilitation Activity subcodes to transcripts for 28 individual and 11 group interviews, as well as 122 site-level debriefing note documents. The analysis team top-coded all documents using the qualitative data analysis software program, Atlas.ti 8. They then sub-coded data for each construct either by reviewing and summarizing the top-level data in a Word document organized by subcodes, or by sub-coding in Atlas.ti; extracting the coded text; and then summarizing data for each subcode. Finally, they compiled the subcode summaries in an Excel document to create a matrix across data sources for each subcode, enabling them to explore similarities and differences across sites as well as interactions between facilitation activities and site characteristics. The analysis team included MJR, KLD, and BNS.

BHIP project researchers piloted the codebook during a secondary analysis of data collected from 31 BHIP providers at nine VA medical centers to understand providers’ perceptions of and experiences with the implementation of the collaborative care model. The goal of the secondary analysis was to examine the implementation barriers and facilitators through the lens of the i-PARIHS framework. They applied codes to interview transcripts for the Innovation, Recipients, and Context constructs, excluding subcodes that were not relevant for their specific collaborative care model implementation effort. For the Context construct, they used the same set of clustered context subcodes applied in the MBC project. Their data sources did not include information about facilitation activities, so the Facilitation activity codes were not used. They applied the top-level construct codes to interview transcripts using NVIVO 12 software and created quote reports for each construct. They then summarized coded text by relevant subcodes for the four i-PARIHS constructs in a Word document. Because they had previously coded the same dataset by collaborative care model elements [42], they also examined the cross-coding between the collaborative care model and i-PARIHS codes/subcodes. The analysis team included JLS, KLD, and another BH QUERI investigator who had served on the expert panel.

After the codebook piloting process was complete for both projects, the analysis teams met to discuss what they had learned that might inform the final version of the codebook. In advance of this meeting, they were asked to review the codes and definitions and consider how they had applied them, whether there were codes they struggled with understanding or applying, whether they edited code definitions, and whether additional instructions were needed for using the codes. They were also asked to provide feedback about the usability of the codebook, advantages and disadvantages to using it, and how we might improve it. We then edited codebook instructions based on their feedback.

## Results

After the preliminary development of the codebook (phase 1), each phase of the development process informed revisions to the results of the previous phase. We thus describe below the results of all four phases for each of the i-PARIHS constructs and for the codebook more generally.

### Innovation

Because the characteristics of the Innovation construct were well described and/or grounded in theory in the core materials, subcode labels were based on characteristics identified by i-PARIHS developers with one exception. Based on literature and our experience, in phase 1, we identified complexity as an important innovation characteristic and added a *Complexity* subcode to the codebook. In phases 2 and 3, the expert panel and i-PARIHS developers concurred. Definitions of the Innovation subcodes were modified throughout all phases of development (see Table 1 for the names of the characteristics of the Innovation construct, the subcode labels/definitions, and modifications made during the development process).

**Table 1:** i-PARIHS Innovation construct characteristics, innovation subcodes, and subcode definitions

Characteristics of the innovation [7]	Innovation subcodes	Innovation subcode definitions
Underlying knowledge sources	Evidence: research/published guidelines	Presence or absence of findings from quantitative, qualitative, or mixed methods studies, as well as literature reviews, that show the efficacy, effectiveness, or other evidence for the innovation (e.g., its utility or acceptability) and also includes a discussion about published guideline recommendations
	Evidence: clinical experience	Presence or absence of professional knowledge of or experience with the innovation which is embedded in or based upon clinical practice and is often tacit and intuitive
	Evidence: patient needs, preferences, and experiences	Presence or absence of patients’ personal knowledge of and experiences with an innovation, including current or previous experiences with the innovation, the extent to which the innovation met/meets their needs and preferences
	Evidence: local practice information	Presence or absence of sources of evidence related to the innovation from the context of care, including, but not limited to, audit and performance data, report cards, progress reports, fidelity ratings, quality improvement and program evaluation data, and financial data/implications
Clarity	Clarity	The degree to which the innovation is understood, including specifics of what components of the innovation must be implemented (for fidelity) and/or what can be adapted or changed
Degree of fit	Degree of fit	The extent to which the innovation is compatible with (1) the values and norms of individuals implementing the innovation and/or (2) the existing practices and operations of the setting, including workflows, processes, roles, and policies
Degree of novelty	Degree of novelty	The extent to which the innovation or components of the innovation are new to or different from individuals’ current thinking, ways of relating to and interacting with each other, or practice
Usability	Usability	The degree of ease or difficulty with which the innovation can be, is, or was adopted and/or used, including the accessibility and availability of information/tools/guides regarding how to adopt/use the innovation
Relative advantage	Relative advantage	Comparison of the innovation with an existing program, practice, or alternative solution and the degree to which one is perceived and/or objectively observed to be more advantageous than the other in meeting patient, clinical, and/or organizational goals and needs
Trialability	Trialability	Whether the innovation can be or has been tested (or experimented with) on a small scale, including discussion about whether it is possible or not possible to conduct a pilot
Observable results	Observable results	The degree to which positive results/benefits of an innovation are directly observable/visible
N/A	Complexity^a^	Ways in which the innovation itself is simple or complicated. Discussion may be about the number of innovation components and/or interaction between them, the number and difficulty of behaviors that those delivering or receiving the innovation must perform, the number of groups or organizational levels targeted by the innovation, and/or the number and variability of outcomes

^a^Subcode added during the codebook development process

### Recipients

There were several challenges to identifying subcodes for the Recipients construct. Although the Innovation and Context constructs were part of the original PARIHS framework, the Recipients construct was new in i-PARIHS and not as well defined conceptually as the others had been. Also, the Recipients construct includes both individuals and teams, making it harder to differentiate some Recipient characteristics from characteristics of the Context. We thus made a number of modifications to subcode labels initially named after the characteristics of recipients identified by developers. First, although i-PARIHS identified motivation, values and beliefs, and goals as distinct Recipient characteristics, we felt it would be hard to parse these out in qualitative material. We therefore opted to combine these characteristics into one subcode, *Personal attributes*; individuals and teams collecting data specifically about the motivation, values and beliefs, and/or goals of study participants can add these for their project-specific codebook. Second, based on i-PARIHS developers’ feedback, we modified the labels for two Context characteristics (time, resources, and support and existing networks) to focus on how the presence or absence of these characteristics affects recipients. Third, at the recommendation of the expert panel, we added the word autonomy to the *Power and authority* subcode label to differentiate the ability to influence the actions of others from recipients’ own actions. Fourth, we did not include local opinion leaders as a subcode because this characteristic delineates a change agent role that may not be applicable for a specific implementation effort, and other types of change agents (e.g., champions, QI team) may be involved. In the codebook, we suggest that subcodes be added for local change agents if capturing change agent roles is important for a study. Finally, during the piloting process, we added a subcode for *General attitude* to account for statements study participants made about what they thought or felt about the innovation generally, e.g., they liked it or enjoyed using it. Definitions of the Recipients subcodes were modified across the phases of development (see Table 2 for the names of the characteristics of the Recipients construct, the subcode labels/definitions, and modifications made during the development process).

**Table 2:** I-PARIHS recipients construct characteristics, recipients subcodes, and subcode definitions

Characteristics of recipients [7]	Recipients subcodes	Recipients subcode definitions
Motivation	Personal attributes^a^	Personal traits or characteristics of any recipient(s). This can include tolerance of ambiguity, general intellectual ability, motivation to change, values, goals, competence, innovativeness, seniority or tenure, learning style, being self-aware, reliable, and other personality traits.
Values and beliefs		
Goals		
Skills and knowledge	Skills and knowledge	What recipients know and understand about the innovation and/or whether recipients have the ability/expertise to perform the tasks required for implementation.
Time, resources, support	How time, resources, and support affect recipients^b^	How the presence or absence of sufficient time, resources, and support is affecting/affected by the ability of a specific recipient (individual or team) to implement or receive the innovation.
Collaboration and teamwork	Collaboration and teamwork	Group processes and team-related issues, including the presence or absence of interprofessional collaboration, communication, and teamwork within teams, between teams and managers, and/or between individuals who work together toward a common goal; team building activities; areas of disagreement/conflict between team members or stakeholder groups; and available conflict management/resolution strategies.
Existing networks	How existing networks affect recipients^b^	How formal or informal networks and/or relationships are affecting/affected the ability and/or motivation of a specific recipient (individual or team) to implement or receive the innovation. Networks/relationships may be professional, task-related, or social and may occur at any level or across levels of the context. Examples of formal networks/relationships include memberships, listservs, communities of practice, learning communities, learning collaboratives, and practice-based research networks. Examples of informal networks/relationships include social practices such as getting together with colleagues for lunch, regular hallway conversations with certain colleagues, friendships, and “huddles” among clinical providers/teams.
Power and authority	Power, authority, and autonomy^b^	The capacity or ability of an individual or team to direct or influence their own actions and/or the actions of others. Power and/or authority may be derived from organizational role (e.g., leadership), professional role (e.g., physician, nurse), expertise, relationships to powerful others, and/or ability to offer or deny rewards or use the threat of force to gain compliance.
Presence of boundaries	Presence of boundaries	Experience with boundaries between groups (e.g., professions/occupations, work units, service lines, roles) that influence implementation. Examples include discussion about the lack of communication between primary care and mental health providers and how clinicians’ scopes of practice or discipline/unit-specific restrictions limit the provision of/access to services.
N/A	General attitude^c^	How the interview participant thinks or feels about the innovation generally, e.g., that they like it or do not like it, it is helpful, or they enjoy using it.
Local opinion leaders^d^	N/A	

^a^Motivation, values and beliefs, and goals characteristics of recipients combined under the subcode label Personal attitudes

^b^Names of recipients characteristics modified for subcode label

^c^Subcode added during the development process

^d^Recipients characteristic not included as a subcode; codebook includes recommendations for using this and/or subcodes for other internal change agents as appropriate

### Context

There were also a number of challenges to identifying subcodes for the Context construct. First, i-PARIHS developers identified characteristics of context as unique to particular levels (e.g., policy drivers and priorities are considered characteristics of the outer context). However, in our collective experience, most characteristics of context can/might occur across organizational levels (e.g., local policies and priorities in the inner context might also impact implementation). To address this challenge, we developed two sets of context subcodes, one for characteristics of context and the other for levels of the implementation context (e.g., local, organizational; see Additional file 2 for the full codebook). To develop the context characteristics subcodes, we combined those delineated by the developers if they were similar in content but broken out into different organizational levels. For example, we combined i-PARIHS’s formal and informal leadership support (a characteristic of the inner/local context) and leadership and senior management support (a characteristic of the inner/organizational context) into a single *Leadership support* subcode (see Table 3 for names of the characteristics of the i-PARIHS Context construct, the subcode labels/definitions, and modifications made during the development process). In the codebook, we recommend that characteristics of context be co-coded with the level(s) at which they occur. For example, to code discussion of the influence of a broad organizational policy affecting multiple units in a large healthcare organization, coders would apply both the *Policies and priorities* code for the characteristic of the context and the *Inner/Organizational* code to identify the level of context.

**Table 3:** i-PARIHS context construct characteristics, context subcodes, and definitions

Characteristics of context by level [7]	Context subcodes	Context subcode definitions
Inner/local: formal and informal leadership support Inner/organizational: leadership and senior management support	Leadership support	Characteristics or behaviors of formal or informal leaders that either support or interfere with the implementation or sustainment of the innovation. This could include discussion of leadership style, relationship building, role modeling, educating, planning-organizing-aligning, communicating, encouraging, empowering, and/or concrete support, e.g., protected time, space, resources, training. Alternatively, the discussion may be about how leaders fail to provide such support or exhibit negative attitudes/behaviors toward innovation implementation/sustainment.
Inner/local: culture Inner/organizational: culture	Culture and climate^a^	Culture of the organization or organizational unit, including prevailing norms, values, beliefs, meanings, understandings, philosophies, way of life, and assumptions. It also includes discussion about the current climate of the organization or organizational unit, e.g., staff empowerment, morale, attitudes, job satisfaction, and burnout, as well as the degree of stability/instability of the environment in which implementation is occurring/will occur.
Inner/local: past experiences with innovation/change Inner/organizational: history of innovations and change	History of innovation and change	How the organization or organizational unit has historically experienced, undertaken, and responded to past change initiatives and/or innovations.
Inner/local: evaluation and feedback processes	Evaluation, monitoring, and feedback^a^	How the organization or organizational unit collects, assesses, monitors, and disseminates data/information about clinical processes and outcomes, economic outcomes, user experiences, clinical performance, etc. It also includes discussion about data sources (e.g., data dashboards, medical records) and ways in which results are fed back to and used by individuals, teams, and services (e.g., through presentations and/or formal reports). This information may be used, e.g., to understand current ways of working or to improve processes.
Inner/organizational: organizational priorities Outer: policy drivers and priorities and regulatory frameworks	Policies and priorities^a^ (includes mandates)	Organizational policies, policy drivers, mandates, and/or priorities; whether/how these are related to/support/hinder the innovation and/or its implementation; and the changes required. Policies are the decisions, plans, and actions that an organization, organizational unit, state, or country takes to achieve specific goals. They include statements of what needs to happen and how (e.g., legislation enacted by a government, regulations or rules issued to carry out the intent of laws or of regulatory bodies, regulatory frameworks or models for enacting regulations, and organizational policies and procedures). Policy drivers are forces that influence policy decisions, e.g., serious problems, i.e., high rates of suicide; legal or ethical concerns, i.e., lack of equity; and crisis events, i.e., hurricanes and forest fires. Mandates are formal orders/commands/requirements and may be a component of written policies. Organizational priorities are identified areas of focus, e.g., improving access to care and reducing medical errors in healthcare settings.
Inner/organizational: learning networks Outer: interorganizational networks and relationships	Networks and relationships	Formal or informal networks and/or relationships that may be/have been leveraged to support or hinder implementation. Networks/relationships may be professional, task-related, or social and may occur at any level or across levels of the context. Examples of formal networks/relationships include memberships, listservs, communities of practice, learning communities, learning collaboratives, and practice-based research networks. Examples of informal networks/relationships include social practices such as getting together with colleagues for lunch, regular hallway conversations with certain colleagues, friendships, and “huddles” among clinical providers/teams.
Inner/organizational: structures and systems	Structures and systems	Formal and informal ways in which the organization or organizational unit is structured and managed and/or its processes for accomplishing work. Examples of structure include authority hierarchies (e.g., chain of command), service lines, matrices, specialized or functional units or departments, inter-/multi-disciplinary teams and task forces, and decision-making levels represented in organizational charts. Although structure and systems are not always distinct, systems generally are related to organizational routines and processes, e.g., for information sharing, learning, workflow, and IT.
Inner/organizational: absorptive capacity	Absorptive capacity	How the organization or organizational unit (e.g., department or clinic) identifies, acquires, assimilates, transforms, and/or applies new, valuable knowledge (e.g., evidence, guidelines, best practices). This includes analyzing, processing, interpreting, understanding, combining with existing knowledge, and applying/incorporating new knowledge into organizational competencies and routines.
Outer: incentives and mandates	Incentives and rewards^a^	Mechanisms/strategies that motivate/encourage/reinforce or that deter/discourage the implementation of the innovation and proposed changes, including incentives/rewards (e.g., casual dress day, pizza day, time off, recognition, financial incentives, i.e., pay for performance) and disincentives (e.g., negative performance reviews, reprimands, regulatory requirements).
N/A	Infrastructure, resources, and support^b^	Presence or absence of infrastructure (e.g., facilities, space, equipment, transportation), resources (e.g., funding, staffing, time, education, skills training, materials), and/or support (e.g., supervisory, clerical) for implementing the innovation.
N/A	Political factors and dynamics^b^	Organizational politics, i.e., how individuals or groups use political strategies to gain/use power and/or social influence in order to positively or negatively affect decisions and activities related to the adoption or implementation of an innovation. For example, they might create conflict, form alliances, bargain, use stalling tactics, discredit others, or compromise. If relevant, this code also includes discussion about the larger political environment (e.g., state or national government) and prevailing political ideology (e.g., nationalism, populism) as it relates to innovation implementation.
Inner/local: mechanisms for embedding change^c^	N/A	
Outer: environmental stability^c^	N/A	

^a^Context characteristic names modified for subcode label

^b^Subcode added during the development process

^c^Context characteristic not given a subcode label; codebook includes recommendations for identifying this higher level concept during the data analysis process

Second, the organizational scientist we consulted felt strongly that there were characteristics of context missing in the framework. To address this concern, we modified some subcodes and added several others. We modified the initial *Culture* subcode by adding the word “climate,” the initial *Evaluation and feedback* subcode by adding the word “monitoring,” and the initial *Incentives* subcode by adding the word “rewards.” For conceptual clarity, we added the word “mandates” to the initial *Policies and priorities* subcode. We also added two new subcodes: *Infrastructure, resources, and support* and *Political factors and dynamics*. As with the other constructs, we modified definitions of the context subcodes across the phases of development.

Finally, several characteristics of Context identified by i-PARIHS, the mechanisms for embedding change and environmental stability are higher-level concepts that can best be identified during the analysis process. For example, i-PARIHS developers suggest that the mechanisms for change may include a variety of activities that fall under other context characteristics, such as regular team meetings and performance review systems (examples of *Structures and systems*) or audit and feedback processes (included in *Evaluation, monitoring, and feedback*). By co-coding characteristics of context subcodes with generic enablers of implementation subcode, data analysts can identify the mechanisms for embedding change during the analysis process. Similarly, environmental stability is related to multiple contextual events/circumstances, e.g., changes or lack of changes in structures and systems, leadership, or policies and procedures. We felt that ultimately determining environmental stability calls for judgment based on a full understanding of the context at multiple levels. In the codebook, we suggest that these two characteristics of context be explored during later stages of data analysis.

### Facilitation activities

We based the initial Facilitation Activity subcodes on 32 activities previously identified and defined [33, 34], and we clustered these into 10 higher-order facilitation activity subcodes. Based on expert panel recommendations, we standardized subcode labels (e.g., *Providing clinical education* rather than *Clinical education*), made significant changes to many of the individual subcode definitions, and made minor changes to the clustered subcode definitions (see Table 4 for the clustered subcodes and final definitions; see Additional file 2 for the 32 individual subcodes and definitions).

**Table 4:** Facilitation activities clustered subcodes, definitions, and individual subcodes

Clustered subcodes	Facilitation activities clustered subcode definitions and individual subcodes^**a**^
Providing education/information	Educating stakeholders on clinical skills, the conduct of innovation marketing, and/or organizational change processes and providing information to promote/publicize the innovation. This includes (1) the content of education/information (e.g., information about the innovation and evidence for it, reasons for change, potential outcomes, clinical knowledge/skills needed) and/or (2) the process of providing education/information (e.g., teaching, training, mentoring, coaching, supervision, experiential/active learning). *This is a cluster code that can be subcoded with the following activity codes: providing education on clinical skills, providing education on marketing, providing education on organizational change, and marketing.*
Collecting data/providing feedback	Collecting data and other information to (1) assess and understand the local context, baseline performance, and implementation barriers/enablers; (2) collect/monitor implementation activities, progress, and outcomes; and (3) provide stakeholders with feedback on data and updates on implementation activities and relevant professional or system-level information. *This is a cluster code which can be subcoded with the following activity codes: conducting ongoing monitoring of innovation implementation, data collection to assess context and baseline performance, and providing updates and feedback.*
Building relationships, teams, and networks	Engaging and building relationships with stakeholders, seeking their participation and buy-in, overcoming resistance to change, managing groups and team processes (including creating an atmosphere of mutual respect, empowering group members, and building relationships between them), and fostering stakeholder networking with peers and external experts/organizations. *This is a cluster code that can be subcoded with the following activity codes: engaging stakeholders, obtaining buy-in, fostering networking with experts, fostering peer networking, managing group/team processes, and overcoming resistance to change.*
Enabling/fostering change	Encouraging, promoting, and helping to support changes in the organization, including interceding and liaising with leadership or other stakeholders and assisting with the development of strategies and policies. The target of change efforts may be the organizational structure or culture or the target of change may not be specified but the methods of fostering change are specified. (For example, the discussion may be about assisting stakeholders with conducting quality improvement activities, helping them build capacity for sustainment, or guiding and supporting them during the implementation process.) *This is a cluster code that can be subcoded with the following activity codes: fostering organizational change: cultural; fostering organizational change: structural; fostering change/unspecified; interceding/liaising with others; and strategy/policy development.*
Problem identification and resolution	Conducting or helping stakeholders (1) identify, become aware of, or clarify implementation challenges/barriers/problems and/or (2) generate potential solutions/countermeasures or select the one(s) most likely to address/solve implementation challenges/barriers/problems. *This is a cluster code and can be subcoded with the following activity codes: problem identification and problem solving.*
Planning/preparing for implementation	Helping stakeholders develop or refine action/implementation plans, come to a consensus, adapt the innovation to the local context (structure, staffing, culture, and other initiatives), share a vision for change, and identify goals and priorities. *This is a cluster code that can be subcoded with the following activity codes: action/implementation planning, adapting innovation to the local context, developing shared vision/consensus building, and goal/priority setting.*
Helping to define, identify, and fill stakeholder roles	Helping to identify and select local change agents (e.g., facilitators, QI team members, local champions, opinion leaders) and/or hire innovation providers, as well as establish, describe/clarify, and/or allocate facilitator and stakeholder roles and responsibilities. *This is a cluster code and can be subcoded with the following activity codes: describing/clarifying roles and responsibilities, helping to hire clinical program staff, and helping identify/select local change agents.*
Providing administrative/technical support	Conducting administrative tasks that support the operationalization of implementation activities and providing technical support, i.e., practical help and assistance to support implementation. Examples of administrative tasks include arranging calls, meetings, and implementation site visits; developing/preparing and disseminating minutes/reports and educational/marketing materials; and organizing innovation provider training. Examples of technical support include providing tools/sample materials; working with site stakeholders to co-create tools/materials, identifying/providing information about available resources for implementation, and working with relevant stakeholders to ensure that information technology (IT) systems accurately capture innovation activity and support implementation. *This is a cluster code and may be subcoded with the following activity codes: administrative tasks and technical support*.
Using interpersonal skills to create a supportive environment	Using positive, supportive behaviors and communications to create an open, supportive, and trusting environment conducive to change, including being generally helpful and available, communicating regularly, acknowledging ideas and efforts, and celebrating achievements/success. This code also includes selectively reducing the level of facilitation support, including positive supportive behaviors, in order to allow the transfer of facilitation roles to site stakeholders. *This is a cluster code that can be subcoded with the following activity codes: providing support and pulling back/transferring roles.*
Obtaining/disseminating innovation or facilitation knowledge	Obtaining information about/developing skills needed for facilitating the implementation of the innovation or fostering dissemination of knowledge about the innovation or facilitation other than at the implementation site(s). Facilitators may foster dissemination by attending, presenting at, or organizing non-local meetings or by assisting with dissemination at sites not receiving facilitation. *This is a cluster code that can be subcoded with the following activity codes: attending, presenting at, and/or organizing non-local meetings; fostering the spread of clinical innovation/facilitation methods; and obtaining training/continuing education.*

^a^See Additional file 2 for definitions of individual subcodes

### Instructions and explanatory material

In addition to i-PARIHS construct codes, subcodes for the characteristics of constructs, and definitions, the codebook (see Additional file 2) includes instructions for the application of individual codes and for the use of the codebook more generally. We initially developed instructions for applying some of the individual codes during phase 1 and then modified them based on feedback from the expert panel, i-PARIHS experts, and the piloting process. Instructions expand on subcode definitions by providing inclusion and/or exclusion criteria, as well as guidance for differentiating between subcodes and/or co-coding when appropriate or subcodes are difficult to differentiate.

We also developed instructions for the use of the codebook, as well as explanatory material. For example, we encourage users to adapt the codebook to their projects and to create their own examples. We also recommend that they co-code i-PARIHS subcodes, as applicable, to indicate factors that may impede implementation (barriers) and/or factors that may enhance or improve implementation (enablers). We added instructions and explanatory material based on feedback across the phases of development. For example, in response to i-PARIHS developers’ recommendations, we clarified that we developed the codebook based on our interpretation of the i-PARIHS framework.

## Discussion

The lack of standardized definitions of the i-PARIHS framework’s constructs and sub-constructs had resulted in the development of definitions for individual studies and limited the opportunity for comparing findings across studies that are guided by i-PARIHS. Using a rigorous, structured process, the authors developed a qualitative codebook, informed by the i-PARIHS framework, that addresses this gap. When developing the codebook, our goal was to stay as close as possible to the intent and purpose of the i-PARIHS framework. We also focused on the demands of qualitative data analysis, the challenges of assigning a priori codes to what people say during interviews, and the needs of coding teams. Thus, we sought to ensure that code labels were conceptually meaningful and clear and that definitions and instructions could be understood and applied by individuals with or without experience using i-PARIHS. Developers of the i-PARIHS framework, as well as expert panel members, asserted that the codebook is a good resource for researchers.

We do not consider the codebook a significant revision to i-PARIHS. Rather, it is a tool, a codebook informed by i-PARIHS, for individuals and teams conducting qualitative data analysis to identify and describe what has influenced innovation implementation. For the Innovation, Recipients, and Context constructs, the codebook added several subcodes for characteristics that are consistent with i-PARIHS, but not specifically mentioned, and modified the names of several others so that subcode labels were clear and inclusive. The i-PARIHS framework did not include a sub-classification of facilitation activities but did suggest how facilitators could address the characteristics of the other three constructs. We adopted and revised a list of facilitation activities from previous work that included an extensive literature review, a qualitative study of an intensive facilitation strategy conducted over two and a half years, and a scoping review of the literature. The list and definitions we adopted were informed by the PARIHS framework and our adaptations during the codebook development process were informed by literature available about the i-PARIHS framework. Thus, it is not surprising that subcodes for clustered facilitation activities and individual activities are similar to actions recommended by the framework. For example, i-PARIHS recommends goal setting and consensus building; the codebook includes subcodes for *Setting goals and priorities* and *Developing shared vision/consensus building*. It is likely that some qualitative material will need to be co-coded for multiple facilitation activities. For example, material co-coded with subcodes from the *Building relationships, teams, and networks* and *Enabling and fostering change* facilitation activities clusters may be descriptions of the i-PARIHS boundary spanning concept which consists of scanning the organizational environment and employing four different types of activities: collecting and disseminating information; developing and maintaining relationships; coordinating, aligning, and negotiating with the environment; and facilitating cooperation by mediating between different interests and identities [43, 44].

Although i-PARIHS Facilitation construct recommendations are linked to other specific constructs (e.g., problem identification is linked only to the Innovation construct), the codebook will allow users to co-code qualitative material without assuming that particular characteristics are linked (e.g., material coded with the *Problem* identification subcode might be co-coded with one or more of the subcodes for characteristics of the context). Additionally, although characteristics of the i-PARIHS context construct were targeted to specific levels of context (e.g., formal and informal leadership support is only a characteristic of the inner/local context), the codebook allows users to code which levels of context qualitative material are describing. Unlike actions recommended in the Facilitation guide [7], subcodes in the codebook are not linked a priori to specific other construct subcodes. Codebook users will be able to cross-code activities with other construct subcodes in order to explore how facilitators actually address barriers and leverage enablers related to construct characteristics, as well as how these constructs interact with each other.

Theories, models, and frameworks, i.e., i-PARIHS, are often treated as “received wisdom” that can inform research [45] with less emphasis on using research findings iteratively and recursively to further develop, refine, and expand them [46]. Developers of the original framework [8] continued to refine PARIHS by conducting literature reviews and concept analyses [9, 47, 48], exploring evidence on users’ application of the framework [37], and continuing to develop and refine how the framework could be used [10]. Two PARIHS developers continued this work by reviewing the evidence on users’ experiences with PARIHS, reviewing critiques of PARIHS, and comparing PARIHS to other frameworks and models. As a result, they revised the original framework, which is now called i-PARIHS. This history of refinement will likely continue. In addition to focusing analysis of data for individual studies, the codebook described in this paper can support the refinement of the i-PARIHS framework using empirical findings from multiple studies. Studies applying the codebook can test existing constructs and proposed relationships between their characteristics, as well as characteristics represented by subcodes we added (e.g., expansion of context codes to all levels, inclusion of additional context codes, and how facilitation activities are used to address characteristics of other constructs). CFIR framework developers have recently revised some of that framework’s constructs, sub-constructs, and definitions based on a review of literature and a survey of authors who had applied CFIR in a published study [49]. In the future, i-PARIHS developers or others might use similar methods to further refine i-PARIHS based on evidence from studies that have applied the codebook.

### Limitations

There are several limitations to the development of the codebook. First, for the Facilitation construct, we focused exclusively on the activities that facilitators conduct. Facilitator skills and attributes can impact both the types and quality of activities facilitators conduct, e.g., lack of appropriate skills can potentially have a negative impact on fidelity to a planned facilitation strategy and ultimately on outcomes [50–53]. We acknowledge that evaluation of an implementation facilitation intervention should likely include an assessment of facilitators’ skills, but the addition of sub-codes for skills was beyond the scope of the workgroup. However, previous work, which identified and described facilitation skills [54], could form the foundation for coding facilitation skills in qualitative material. Future work should apply these and further refine descriptions for addition to the current codebook.

Second, we piloted the codebook on data collected using interview guides that were broadly informed by i-PARIHS constructs but not designed to probe for the specific construct characteristics represented by subcodes. The coding of data thus resulted in the identification of construct characteristics that were most salient to study participants and confirmed that the codebook could be applied to data. Future studies should develop and apply interview guides that include probes for construct characteristics and test the codebook to maximize its value. Finally, the codebook was developed based on the authors’ experiences conducting qualitative research and applying the PARIHS and/or i-PARIHS frameworks. Similar to developers of the CFIR, the authors are researchers embedded in the VA [49]. Just as the CFIR has been widely utilized both within and outside of VA [55], we believe that the codebook we developed is also widely applicable. It is important to note that although our team created the codebook independently, i-PARIHS developers reviewed it and their input was incorporated into the final version.

## Conclusions

This paper describes the development of a qualitative codebook informed by the i-PARIHS framework. The codebook can focus the analysis of data for individual studies, and facilitate data exploration, pattern identification, and insight development which are critical for meaningful use of TMFs in implementation studies. It also provides standardized subcodes and definitions for each of the i-PARIHS constructs and construct characteristics, maximizing the potential for comparing findings across studies informed by i-PARIHS, and advancing generalizable implementation science knowledge. Using the codebook will enable qualitative data analysts to explore interactions between the innovation, recipients, and context and how facilitators interact with these. Finally, the codebook described in this paper can support the refinement of the i-PARIHS framework using empirical findings from multiple studies.

## Supplementary Information

**Additional file 1.** Literature that informed development of the i-PARHIS codebook. Literature WG members reviewed to identify and define subcodes for i-PARIHS construct characteristics.

**Additional file 2.** A qualitative codebook informed by the i-PARIHS framework. Qualitative codebook, informed by the i-PARIHS framework, that includes sub-codes and definitions for each of the framework’s constructs and instructions for using the codebook.

## Data Availability

Not applicable.

## Abbreviations

BH QUERI: Behavioral Health Quality Enhancement Research Initiative
CFIR: Consolidated Framework for Implementation Research
PARIHS: Promoting Action on Implementation Research in Health Services
i-PARIHS: Integrated-Promoting Action on Implementation Research in Health Services
TMFs: Theories, models, and frameworks
VA: Department of Veterans Affairs
