# Supplementary material for: Development of a qualitative data analysis codebook informed by the i-PARIHS framework
Source: Implement Sci Commun. 2022 Sep 14;3:98. doi: 10.1186/s43058-022-00344-9 (PMC9476709; doi:10.1186/s43058-022-00344-9)
Supplement: Supplementary file 1 — Additional file 1. Literature that informed development of the i-PARHIS codebook. Literature WG members reviewed to identify and define subcodes for i-PARIHS construct characteristics. [file 43058_2022_344_MOESM1_ESM.docx]

**Literature that informed development of subcode definitions for the Innovation, Recipients, and Context constructs**

Workgroup members reviewed and discussed i-PARIHS source material [1, 2], their own knowledge and experience, other relevant literature, and feedback from experts and pilot participants to develop the Innovation, Recipients, and Context subcodes and definitions. The tables below cite the literature workgroup members reviewed for each of the characteristics of these i-PARIHS constructs.

| **Innovation** | |
| --- | --- |
| Evidence | [3-5] |
| Clarity | [6] |
| Degree of fit | [7, 8] |
| Degree of novelty | No other literature |
| Usability | [7, 9] |
| Relative advantage | [10] |
| Trialability | [7, 10] |
| Observable results | [7] |
| Complexity | [7, 11-15] |

| **Recipients** | |
| --- | --- |
| Personal attributes | [10, 16-20] |
| Skills and knowledge | No other literature |
| How time, resources, and support affect recipients | [10] |
| Collaboration and teamwork | [10, 21-30] |
| How existing networks affect recipients | [21-23, 25, 31-33] |
| Power, authority, and autonomy | [34-39] |
| Presence of boundaries | [32, 40-47] |
| General attitude | Informed by codebook pilot |

| **Context** | |
| --- | --- |
| Leadership support | [48, 49] |
| Culture and climate | [10, 49-52] |
| History of innovation and change | [33] |
| Evaluation, monitoring, and feedback | [10] |
| Policies and priorities | [53-61] |
| Networks and relationships | [10, 21-30] |
| Structures and systems | [62-65] |
| Absorptive capacity | [66-69] |
| Incentives and rewards | [10, 22, 25, 49, 70, 71] |
| Infrastructure, resources, and support | No other literature |
| Political factors and dynamics | [72-78] |

References

1. Harvey G, Kitson A. Implementing evidence-based practice in healthcare: a facilitation guide. London: Routledge; 2015.

2. Harvey G, Kitson A. PARIHS revisited: from heuristic to integrated framework for the successful implementation of knowledge into practice. Implement Sci*.* 2016;11(1):1-13. <https://doi.org/10.1186/s13012-016-0398-2>

3. Rycroft-Malone J, Seers K, Titchen A, Harvey G, Kitson A, McCormack B. What counts as evidence in evidence-based practice? J Adv Nurs*.* 2004;47(1):81-90. <https://doi.org/10.1111/j.1365-2648.2004.03068.x>

4. Helfrich C, Damschroder L, Hagedorn H, Daggett G, Sahay A, Ritchie M, et al. A critical synthesis of literature on the Promoting Action on Research Implementation in Health Services (PARIHS) framework. Implement Sci*.* 2010;5(1):82. <https://doi.org/10.1186/1748-5908-5-82>

5. Stetler CB, Damschroder LJ, Helfrich CD, Hagedorn HJ. A Guide for applying a revised version of the PARIHS framework for implementation. Implement Sci*.* 2011;6(1):99. <https://doi.org/10.1186/1748-5908-6-99>

6. Lyons TF. Role clarity, need for clarity, satisfaction, tension, and withdrawal. Organ Behav Hum Perform*.* 1971;6(1):99-110. <https://doi.org/10.1016/0030-5073(71)90007-9>

7. Rogers EM. Diffusion of innovations. 5th ed. New York: Free Press; 2003.

8. Tornatzky LG, Klein KJ. Innovation characteristics and innovation adoption-implementation: a meta-analysis of findings. IEEE Trans Eng Manag*.* 1982;EM-29(1):28-45. <https://doi.org/10.1109/TEM.1982.6447463>

9. Rogers EM. Diffusion of innovations. 3rd ed. New York: Free Press; 1983.

10. Damschroder LJ, Aron DC, Keith RE, Kirsh SR, Alexander JA, Lowery JC. Fostering implementation of health services research findings into practice: a consolidated framework for advancing implementation science. Implement Sci*.* 2009;4(1):50. <https://doi.org/10.1186/1748-5908-4-50>

11. Craig P, Dieppe P, Macintyre S, Michie S, Nazareth I, Petticrew M. Developing and evaluating complex interventions: the new Medical Research Council guidance. BMJ*.* 2008;337:a1655. <https://doi.org/10.1136/bmj.a1655>

12. Craig P, Dieppe P, Macintyre S, Michie S, Nazareth I, Petticrew M. Developing and evaluating complex interventions: the new Medical Research Council guidance. Int J Nurs Stud*.* 2013;50(5):587-92. <https://doi.org/10.1016/j.ijnurstu.2012.09.010>

13. Pfadenhauer L, Rohwer A, Burns J, Booth A, Lysdahl KB, Hofmann B, et al. Guidance for the assessment of context and implementation in health technology assessments (HTA) and systematic reviews of complex interventions: the context and implementation of complex interventions (CICI) framework. 2016. <https://scholar.google.de/citations?view_op=view_citation&hl=en&user=XZEqKDIAAAAJ&citation_for_view=XZEqKDIAAAAJ:2osOgNQ5qMEC>. Accessed 31 Aug 2018.

14. Pfadenhauer LM, Gerhardus A, Mozygemba K, Lysdahl KB, Booth A, Hofmann B, et al. Making sense of complexity in context and implementation: the Context and Implementation of Complex Interventions (CICI) framework. Implement Sci*.* 2017;12(1):21. <https://doi.org/10.1186/s13012-017-0552-5>

15. Medical Research Council . Health Services Public Health Research Board: A framework for development and evaluation of RCTs for complex interventions to improve health. In*.*: Medical Research Council; 2000. <https://mrc.ukri.org/documents/pdf/rcts-for-complex-interventions-to-improve-health/>. Accessed 31 Aug 2018.

16. Cranley LA, Cummings GG, Profetto-McGrath J, Toth F, Estabrooks CA. Facilitation roles and characteristics associated with research use by healthcare professionals: a scoping review. BMJ Open*.* 2017;7(8). <https://doi.org/10.1136/bmjopen-2016-014384>

17. Flottorp SA, Oxman AD, Krause J, Musila NR, Wensing M, Godycki-Cwirko M, et al. A checklist for identifying determinants of practice: a systematic review and synthesis of frameworks and taxonomies of factors that prevent or enable improvements in healthcare professional practice. Implement Sci*.* 2013;8:35. <https://doi.org/10.1186/1748-5908-8-35>

18. Michie S, Johnston M, Abraham C, Lawton R, Parker D, Walker A, et al. Making psychological theory useful for implementing evidence-based practice: a consensus approach. Qual Saf Health Care*.* 2005;14:26-33. <https://doi.org/10.1136/qshc.2004.011155>

19. Gilley A, Gilley JW, McMillan HS. Organizational change: motivation, communication, and leadership effectiveness. Performance Improvement Quarterly*.* 2009;21(4):75-94. <https://doi.org/10.1002/piq.20039>

20. Kalling T. Organization-internal transfer of knowledge and the role of motivation: a qualitative case study. Knowledge and Process Management*.* 2003;10(2):115-26. <https://doi.org/10.1002/kpm.170>

21. Harvey G, Fitzgerald L, Fielden S, McBride A, Waterman H, Bamford D, et al. The NIHR Collaboration for Leadership in Applied Health Research and Care (CLAHRC) for Greater Manchester: combining empirical, theoretical and experiential evidence to design and evaluate a large-scale implementation strategy. Implement Sci*.* 2011;6(1):1-12. <https://doi.org/10.1186/1748-5908-6-96>

22. Harvey G, Kitson A. Translating evidence into healthcare policy and practice: single versus multi-faceted implementation strategies − is there a simple answer to a complex question? Int J Health Policy Manag*.* 2015;4(3):123-6. <https://dx.doi.org/10.15171%2Fijhpm.2015.54>

23. Greenhalgh T, Robert G, MacFarlane F, Bate P, Kyriakidou O. Diffusion of innovations in service organizations: systematic review and recommendations. Millbank Q*.* 2004;82(4):581-629. <https://doi.org/10.1111/j.0887-378X.2004.00325.x>

24. Grol RP, Bosch MC, Hulscher M, Eccles MP, Wensing M. Planning and studying improvement in patient care: the use of theoretical perspectives. Millbank Q*.* 2007;85(1):93-138. <https://dx.doi.org/10.1111%2Fj.1468-0009.2007.00478.x>

25. Kitson A, Brook A, Harvey G, Jordan Z, Marshall R, O'Shea R, et al. Using complexity and network concepts to inform healthcare knowledge translation. Int J Health Policy Manag*.* 2018;7(3):231-43. <https://dx.doi.org/10.15171%2Fijhpm.2017.79>

26. Cunningham FC, Ranmuthugala G, Plumb J, Georgiou A, Westbrook JI, Braithwaite J. Health professional networks as a vector for improving healthcare quality and safety: a systematic review. BMJ Qual Saf*.* 2012;21(3):239-49. <https://doi.org/10.1136/bmjqs-2011-000187>

27. Poghosyan L, Lucero RJ, Knutson AR, W. Friedberg M, Poghosyan H. Social networks in health care teams: evidence from the United States. J Health Organ Manag*.* 2016;30(7):1119-39. <https://doi.org/10.1108/JHOM-12-2015-0201>

28. Reeves S, Xyrichis A, Zwarenstein M. Teamwork, collaboration, coordination, and networking: why we need to distinguish between different types of interprofessional practice. J Interprof Care*.* 2018;32(1):1-3. <https://doi.org/10.1080/13561820.2017.1400150>

29. Xyrichis A, Reeves S, Zwarenstein M. Examining the nature of interprofessional practice: an initial framework validation and creation of the InterProfessional Activity Classification Tool (InterPACT). J Interprof Care*.* 2018;32(4):416-25. <https://doi.org/10.1080/13561820.2017.1408576>

30. Borgatti SP, Foster PC. The network paradigm in organizational research: a review and typology. J Manag*.* 2003;29(6):991-1013. <https://doi.org/10.1016/S0149-2063(03)00087-4>

31. Harvey G, Lynch E. Enabling continuous quality improvement in practice: the role and contribution of facilitation. Front Public Health*.* 2017;5:27. <https://doi.org/10.3389/fpubh.2017.00027>

32. Carlile PR. Transferring, translating, and transforming: an integrative framework for managing knowledge across boundaries. Organization Science*.* 2004;15(5):555-68. <https://doi.org/10.1287/orsc.1040.0094>

33. Weiner BJ. A theory of organizational readiness for change. Implement Sci*.* 2009;4(1):67. <https://doi.org/10.1186/1748-5908-4-67>

34. Rycroft-Malone J. The PARIHS Framework-a framework for guiding the implementation of evidence-based practice. J Nurs Care Qual*.* 2004;19(4):297-304. <https://doi.org/10.1097/00001786-200410000-00002>

35. Etzioni A. A comparative analysis of complex organizations: on power, involvement and their correlates. New York: Free Press; 1961.

36. Erasmus E, Gilson L. How to start thinking about investigating power in the organizational settings of policy implementation. Health Policy Plan*.* 2008;23(5):361-8. <https://doi.org/10.1093/heapol/czn021>

37. Rycroft-Malone J, Seers K, Chandler J, Hawkes CA, Crichton N, Allen C, et al. The role of evidence, context, and facilitation in an implementation trial: implications for the development of the PARIHS framework. Implement Sci*.* 2013;8:28. <https://doi.org/10.1186/1748-5908-8-28>

38. Raven BH. The bases of power: origins and recent developments. J Soc Issues*.* 1993;49(4):227-51. <https://doi.org/10.1111/j.1540-4560.1993.tb01191.x>

39. Elmore RF. Backward mapping: implementation research and policy decisions. Political Science Quarterly*.* 1979;94(4):601-16. <https://doi.org/10.2307/2149628>

40. Currie G, White L. Inter-professional barriers and knowledge brokering in an organizational context: the case of healthcare. Organ Stud*.* 2012;33(10):1333-61. <https://doi.org/10.1177%2F0170840612457617>

41. Rycroft-Malone J, Harvey G, Seers K, Kitson A, McCormack B, Titchen A. An exploration of the factors that influence the implementation of evidence into practice. J Clin Nurs*.* 2004;13(8):913-24. <https://doi.org/10.1111/j.1365-2702.2004.01007.x>

42. Ashkenas R, Ulrich D, Jick T, Kerr S. The boundaryless organization: breaking the chains of organizational structure. 2nd ed. San Francisco: Jossey-Bass; 2002.

43. Rodríguez C, Langley A, Béland F, Denis J. Managing across boundaries in health care: the forces of change and inertia. In: Paulsen N, Hernes T, editors. Managing boundaries in organizations: multiple perspectives. London: Palgrave Macmillan; 2003. p. 147-68.

44. Paulsen N. "Who are we now?": group identity, boundaries, and the (re)organizing process. In: Paulsen N, Hernes T, editors. Managing boundaries in organizations: multiple perspectives. London: Palgrave Macmillan; 2003. p. 14-34.

45. Tasselli S. Social networks and inter-professional knowledge transfer: the case of healthcare professionals. Organ Stud*.* 2015;36(7):841-72. <https://doi.org/10.1177%2F0170840614556917>

46. Prætorius T, Becker MC. How to achieve care coordination inside health care organizations: insights from organization theory on coordination in theory and in action. Int J Care Coord*.* 2015;18(4):85-92. <https://doi.org/10.1177%2F2053434516634115>

47. Kislov R, Walshe K, Harvey G. Managing boundaries in primary care service improvement: a developmental approach to communities of practice. Implement Sci*.* 2012;7:97-. <https://doi.org/10.1186/1748-5908-7-97>

48. Stetler CB, Ritchie JA, Rycroft-Malone J, Charns MP. Leadership for evidence-based practice: strategic and functional behaviors for institutionalizing EBP. Worldviews Evid Based Nurs*.* 2014;11(4):219-26. <https://doi.org/10.1111/wvn.12044>

49. Kitson A, Harvey G, McCormack B. Enabling the implementation of evidence based practice: a conceptual framework. Qual Health Care*.* 1998;7(3):149-58. <https://doi.org/10.1136/qshc.7.3.149>

50. Aarons GA, Hurlburt M, Horwitz SM. Advancing a conceptual model of evidence-based practice implementation in public service sectors. Adm Policy Ment Health*.* 2011;38(1):4-23. <https://doi.org/10.1007/s10488-010-0327-7>

51. Denison DR. What is the difference between organizational culture and organizational culture and organizational climate? A native's point of view on a decade of paradigm wars. Acad Manage Rev*.* 1996;21(3):619-54. <https://doi.org/10.5465/amr.1996.9702100310>

52. Schein EH. Organizational culture and leadership. 3rd ed. San Francisco: Jossey-Bass; 2004.

53. Wensing M, Eccles M, Grol R. Economic and policy strategies for implementation of change. In: Grol R, Wensing M, Eccles M, David D, editors. Improving Patient Care. Chichester, West Sussex: Wiley-Blackwell; 2013. p. 240-53.

54. Kilpatrick DG. Definitions of public policy and the law. <https://mainweb-v.musc.edu/vawprevention/policy/definition.shtml>. Accessed: 15 Sep 2018.

55. Rütten A, Gelius P, Abu-Omar K. Policy development and implementation in health promotion—from theory to practice: the ADEPT model. Health Promot Int*.* 2010;26(3):322-9. <https://doi.org/10.1093/heapro/daq080>

56. World Health Organization. Health policy. <https://www.euro.who.int/en/health-topics/health-policy/health-policy>. Accessed: 15 Sep 2018.

57. Peters BG. American public policy. Washington DC: CQ Press, a division of Congressional Quarterly; 2004.

58. Hill M, Hupe P. Implementing public policy: governance in theory and in practice. London: Sage Publications; 2002.

59. Lester P. Policy drivers; putting the politics back in implementation. <http://www.socialinnovationcenter.org/archives/3012>. Accessed: 15 Sep 2018.

60. Hogwood B, Gunn L. Policy analysis for the real world. Oxford: Oxford University Press; 1984.

61. Kingdon JW. Agendas, alternatives, and public policies. 2nd ed. New York: Longman Classics in Political Science; 2003.

62. Tolbert T, Hall R. Organizations: structures, processes and outcomes. 10th ed. Upper Saddle River, N.J.: Pearson Prentice Hall; 2009.

63. Hage J, Aiken M. Social change in complex organizations. New York: Random House; 1970.

64. Evans JM, Grudniewicz A, Gray CS, Wodchis WP, Carswell P, Baker GR. Organizational context matters: a research toolkit for conducting standardized case studies of integrated care initiatives. Int J Integr Care*.* 2017;17(2):9. <https://doi.org/10.5334/ijic.2502>

65. Benzer JK, Charns MP, Hamdan S, Afable M. The role of organizational structure in readiness for change: A conceptual integration. Health Serv Manage Res*.* 2017;30(1):34-46. <https://doi.org/10.1177/0951484816682396>

66. Zahra SA, George G. Absorptive capacity: a review, reconceptualization, and extension. Acad Manage Rev*.* 2002;27(2):185-203. <https://doi.org/10.5465/amr.2002.6587995>

67. Harvey G, Skelcher C, Spencer E, Jas P, Walshe K. Absorptive capacity in a non-market environment: a knowledge-based approach to analysing the performance of sector organizations. Public Manage Rev*.* 2010;12(1):77-97. <https://doi.org/10.1080/14719030902817923>

68. Harvey G, Kitson A. Necessary but not sufficient… comment on "Knowledge mobilization in healthcare organizations: a view from the resource-based view of the firm". Int J Health Policy Manag*.* 2015;4(12):865-8. <https://doi.org/10.15171/ijhpm.2015.159>

69. Ferlie E, Crilly T, Jashapara A, Trenholm S, Peckham A, Currie G. Knowledge mobilization in healthcare organizations: a view from the resource-based view of the firm. Int J Health Policy Manag*.* 2015;4(3):127-30. <https://doi.org/10.15171/ijhpm.2015.35>

70. Kitson AL, Harvey G. Methods to succeed in effective knowledge translation in clinical practice. J Nurs Scholarsh*.* 2016;48(3):294-302. <https://doi.org/10.1111/jnu.12206>

71. Cook JM, O’Donnell C, Dinnen S, Coyne JC, Ruzek JI, Schnurr PP. Measurement of a model of implementation for health care: toward a testable theory. Implement Sci*.* 2012;7(1):59. <https://doi.org/10.1186/1748-5908-7-59>

72. Kirk P, Broussine M. The politics of facilitation. J Workplace Learn*.* 2000;12(1):13-22. <https://doi.org/10.1108/13665620010309756>

73. Mayes BT, Allen RW. Toward a definition of organizational politics. Acad Manage Rev*.* 1977;2(4):672-8. <https://doi.org/10.5465/amr.1977.4406753>

74. Pfeffer J. Understanding power in organizations. Calif Manage Rev*.* 1992;34(2):29-50. <https://doi.org/10.1177%2F000812569203400201>

75. Henning DH. Environmental policy and politics: value and power context. Nat Resour J*.* 1971;11(3):447-54.

76. Schein VE. Political strategies for implementing organizational change. Group & Organization Studies*.* 1977;2(1):42-8. <https://doi.org/10.1177%2F105960117700200106>

77. DuBrin AJ. Political behavior in organizations. Thousand Oaks: Sage Publications; 2009.

78. World Health Organization. Health service planning and policy-making: a toolkit for nurses and midwives. Manila: WHO Regional Office for the Western Pacific; 2005.
