# Supplementary material for: Development of a qualitative data analysis codebook informed by the i-PARIHS framework
Source: Implement Sci Commun. 2022 Sep 14;3:98. doi: 10.1186/s43058-022-00344-9 (PMC9476709; doi:10.1186/s43058-022-00344-9)
Supplement: Supplementary file 2 — Additional file 2. A qualitative codebook informed by the i-PARIHS framework. Qualitative codebook, informed by the i-PARIHS framework, that includes sub-codes and definitions for each of the framework’s constructs and instructions for using the codebook. [file 43058_2022_344_MOESM2_ESM.docx]

**Additional File 2**

**A Qualitative Codebook Informed by the i-PARIHS Framework**

**Purpose**

The original PARIHS and now i-PARIHS framework have been widely used to inform implementation studies, particularly those applying facilitation as an implementation strategy, and to explore factors that influence implementation. The i-PARIHS framework was introduced in the context of a guide for facilitation practitioners [1]. Because this guide lacks formal definitions of the framework’s constructs and construct characteristics that could be used for coding qualitative data, individual studies have developed their own definitions. Lack of standardized definitions limits the opportunity to compare findings across studies and improve our understanding of how these constructs interact. This codebook, informed by the i-PARIHS framework, addresses that gap.

**Overview**

The codebook includes top level codes and definitions for the four constructs of the i-PARIHS framework: Innovation, Recipients, Context, and Facilitation. In the i-PARIHS framework, Innovation, Recipients, and Context are **factors that affect implementation**. The codebook contains subcodes and definitions for the characteristics of these constructs. Facilitation, also a factor that affects implementation is the “**active ingredient in implementation**, working with the core elements of the innovation to be implemented, the recipients of the innovation, and the context in which implementation occurs.” Thus, for the Facilitation construct, subcodes are Facilitation **activities** with **descriptions of what facilitators do** rather than ‘definitions’ of characteristics.

This codebook was developed by a Department of Veterans Affairs (VA) Behavioral Health QUERI workgroup in a rigorous four phase process that included preliminary development tasks, an expert panel process, review by i-PARIHS developers, and preliminary piloting. Innovation, Recipients, and Context subcodes and definitions were predominantly informed by workgroup and expert panel members’ interpretation of original descriptions of the framework, the framework’s theoretical underpinnings, and other relevant literature. I-PARIHS developers concurred with the subcodes and definitions. Because the Facilitation construct lacked defined sub-constructs and activities described in Harvey and Kitson’s book and related article are focused on providing guidance to facilitation practitioners rather than exploring what facilitator do, facilitation activity subcodes were based on literature and previous studies. Users can adopt any or all of the codebook and adapt it for a specific project to understand what influenced implementation. Cross-coding the characteristics of i-PARIHS constructs and facilitation activities will allow investigators to explore interactions between the innovation, the recipients, and the context, as well as between these constructs and what facilitators did.

**Acknowledgements**

VA Behavioral Health QUERI Workgroup members responsible for developing the codebook: Mona J. Ritchie, PhD; Karen L. Drummond, PhD; Sara J. Landes, PhD; Jennifer L. Sullivan, PhD; and Brandy N. Smith, BA. Members of the expert panel: Gregory P. Beehler, PhD; Bo Kim, PhD; JoAnn E. Kirchner, MD; Lindsey A. Martin, PhD; Christopher J. Miller, PhD; Amanda D. Peeples, PhD; Jeffrey L. Smith; Natalie (Kiddie) Vineyard, MS. I-PARIHS developers/experts: Gill Harvey, PhD, Alison Kitson, DPhil, and Sarah Hunter, PhD. Organizational science consultant: Louise Parker, PhD. Codebook development was supported by funding from the Department of Veterans Affairs Quality Enhancement Research Initiative (QUERI), QUE 15-289 and QUE 20-026.

1. Harvey G, Kitson A. Implementing evidence-based practice in healthcare: a facilitation guide. London: Routledge; 2015.

**General instructions**

1. Adapt the codebook to your project

This codebook is designed to be an addition to codes developed for particular projects and it is a *living document*. Codes and definitions informed by the i-PARIHS framework, along with some guidelines (in italics) for using the codes are included. However, users should adapt the codebook for their own needs. For example, subcodes for i-PARIHS construct characteristics are comprehensive but do not all need to be applied. A “menu of constructs” approach can be used to select those constructs literature and theory suggest are most important for implementing a particular innovation. Be sure to add codes to capture emergent themes.

1. Create your own examples

Although the codebook provides instructions for the use of individual subcodes, examples were purposefully not included. Early in the analysis process, codebook users should modify definitions to include project-specific examples and terminology to tailor the codebook for their use.

1. Assume that responses are subjective

Please note that codebook developers assumed that responses to qualitative interview questions are subjective; they are the opinions, thoughts, and perceptions of interviewees. Code definitions thus do not explicitly include this caveat.

1. Use additional “Barriers” and “Enablers” codes

Include a code for “Barriers” and one for “Enablers” so that you can co-code i-PARIHS codes to indicate factors that may impede implementation (barriers) and/or factors that may enhance or improve implementation (enablers).

1. Co-code as needed

Some of the characteristics of i-PARIHS constructs may be difficult to parse out, particularly if the interview guide was not designed to capture these characteristics. For example, in qualitative material, it may be difficult to tell if participants are talking about collaboration and teamwork, a Recipients characteristic, or networks and relationships, a characteristic of the Context. The codebook includes some instructions for co-coding material under such circumstances. Additionally, co-coding for characteristics of constructs will allow you to explore the interactions between constructs.

1. Code other characteristics of the Innovation

It is likely that for your particular study, you will want to code other characteristics of the innovation, e.g., specific and/or core components of the innovation. The i-PARIHS codes are characteristics of innovations that theory and literature suggest may influence implementation.

**Innovation Codes**

The innovation codes include the top-level code and nine subcodes for the characteristics of the innovation that may hinder or enhance implementation. For one of the subcodes, “Evidence/Underlying knowledge sources,” there are four sub-subcodes. When applying these codes, remember that you are coding interview participants’ perceptions, thoughts, and/or understandings about the characteristics of the innovation. Interview participants are often also recipients of the innovation. In this codebook, their perceptions and understandings about the innovation are treated as characteristics of the innovation.

1. **Innovation**

Discussion about the characteristics of the innovation that may influence implementation, including:

- - **Evidence/Underlying knowledge source** for the innovation, including a) research/guidelines, b) clinical experience, c) patient needs/preferences/experiences, and d) local practice information showing innovation outcomes
  - **Clarity** or degree of understanding of what needs to be implemented
  - **Complexity** of the innovation
  - **Degree of fit** (or compatibility) between the innovation and the contextual norms/values/practices/operations
  - **Degree of Novelty** of the innovation
  - **Usability** (or ease) with which the innovation can be used
  - **Relative advantage** (perceived or objective) of using the innovation
  - **Trialability** (or testing) the innovation
  - **Observable Results** of the innovation

**Innovation Subcodes**

1. **Evidence/Underlying knowledge sources**

Discussion about the presence or absence of evidence for the innovation being implemented, including:

- Research/Published Guidelines
- Clinical Experience
- Patient needs, Preferences, and Experiences
- Local Practice Information

1. **Evidence: Research/published guidelines**

Discussion about presence or absence of findings from quantitative, qualitative, or mixed methods studies, as well as literature reviews, that show the efficacy, effectiveness, or other evidence for the innovation (e.g., its utility or acceptability). Also includes discussion about published guideline recommendations.

*Include any discussion about the perceived quality and validity of the studies, reviews, or guidelines.*

1. **Evidence: Clinical experience**

Discussion about presence or absence of professional knowledge of or experience with the innovation which is embedded in or based upon clinical practice and is often tacit and intuitive.

*Include any previous clinical experience with the innovation, e.g., practicums, internships, and previous jobs, and any relevant training in previous jobs or settings. Include discussion about the experiences of others with the innovation, as well as personal practical knowledge.*

1. **Evidence: Patient needs, preferences, and experiences**

Discussion about presence or absence of patients' personal knowledge of and experiences with an innovation, including current or previous experiences with the innovation, the extent to which the innovation met/meets their needs and preferences.

*Includes patients' stories, narratives, and recorded experiences with the innovation, as well as providers’ perceptions and/or reports of patients’ needs, preferences and experiences.*

1. **Evidence: Local practice information**

Discussion about presence or absence of sources of evidence related to the innovation from the context of care, including, but not limited to, audit and performance data, report cards, progress reports, fidelity ratings, quality improvement and program evaluation data, and financial data/implications.

*Include sources of evidence from the site implementing the innovation or information from other sites.*

1. **Clarity**

Discussion about the degree to which the innovation is understood, including specifics of what components of the innovation must be implemented (for fidelity) and/or what can be adapted or changed.

*Exclude statements that are about the interview participant’s opinion about the value of the innovation and/or whether it should be implemented. Note that innovation clarity is related to how easy or hard it is to understand the innovation; innovation usability, another characteristic of innovations, is related to how easy or hard it is to use the innovation.*

1. **Complexity**

Discussion about the ways in which the innovation itself is simple or complicated. Discussion may be about the number of innovation components and/or interaction between them, the number and difficulty of behaviors that those delivering or receiving the innovation must perform, the number of groups or organizational levels targeted by the innovation, and/or the number and variability of outcomes.

*Exclude statements regarding the intricacy/complexity or simplicity of implementing the innovation (i.e., the implementation process) and code with appropriate other i-PARIHS codes. Co-code with “Usability” if interview participant also talks about the difficulty of using the innovation. Co-code with “Clarity” if interview participant also talks about how hard it is to understand the innovation.*

1. **Degree of fit**

Discussion about the extent to which the innovation is compatible with 1) the values and norms of individuals implementing the innovation and/or 2) the existing practices and operations of the setting, including workflows, processes, roles, policies, etc.

*When coding the degree of fit with adopters' norms, values, beliefs, etc., co-code with the Recipients code(s), if appropriate. For example, if discussion includes information about the adopter’s specific values or beliefs, not just that they fit with the innovation, co-code with the “Personal attributes” Recipient code. When coding degree of fit with current practices and operations, co-code with Context code(s) if appropriate. For example, if discussion includes information about how the innovation fits with specific local policies, co-code with “Level/Inner local context” and “Context: Policy and priorities.”*

1. **Degree of novelty**

Discussion about the extent to which the innovation or components of the innovation is/are new to or different from individuals’ current thinking, ways of relating to and interacting with each other, or practice.

*If discussion is also about how the innovation is more or less advantageous than current thinking, ways of relating, or practice, co-code with “Relative Advantage.”*

1. **Usability**

Discussion about the degree of ease or difficulty with which the innovation can be, is, or was adopted and/or used, including the accessibility and availability of information/tools/guides regarding how to adopt/use the innovation.

*Note that innovation usability is related to how easy or hard it is to use the innovation; innovation clarity is related to how easy or hard it is to understand the innovation. Note also that usability may be related to degree of novelty; cross-code when appropriate. For example, if the interview participant is discussing the fact that the innovation is hard to use because it requires providers to relate to each other in new ways, co-code with both usability and degree of novelty.*

1. **Relative advantage**

Discussion comparing the innovation with an existing program, practice, or alternative solution and the degree to which one is perceived and/or objectively observed to be more advantageous than the other in meeting patient, clinical, and/or organizational goals and needs.

*Include statements that describe the innovation as better (or worse than) existing programs. If the discussion is comparing the advantages of objectively observed results of the innovation, co-code with “Observable results.”*

1. **Trialability**

Discussion about whether the innovation can be or has been tested (or experimented with) on a small scale, including discussion about whether it is possible or not possible to conduct a pilot.

*For example, discussion may be about using PDSA cycles for local improvement initiatives. If participant is describing the use of results only from local or regional pilots, consider coding instead with “Underlying knowledge source: Local Practice Information.”*

1. **Observable results**

Discussion about the degree to which positive results/benefits of an innovation are directly observable/visible.

*Co-code with “Relative advantage” when discussion is also about how the results of the innovation are more advantageous than those of an existing program, practice, or solution.*

**Recipient Codes**

The recipient codes include the top-level code and eight subcodes for the characteristics of the individuals and teams (who are the target of the innovation) that may hinder or enhance implementation. According to i-PARIHS, these characteristics influence recipients’ motivations and/or abilities which then influences implementation of the innovation.

*Note: The i-PARIHS framework includes local opinion leaders as a characteristic of recipients. We did not include this as a subcode in the list below. A local opinion leader is a change agent role that may be applicable for a specific implementation effort; however, other change agents (e.g., champions, QI team members) may also be involved. We recommend that you add subcodes for such local change agents if capturing their roles is important for your study.*

**R. Recipients**

Discussion about the characteristics of the targets (individuals and/or teams/groups) of the innovation and/or implementation effort that may influence implementation, including:

- - **Personal attributes** of recipients
  - **Skills and knowledge** of recipients
  - **How time, resources, and support** affect recipients
  - **Collaboration and teamwork** among recipients
  - **How existing networks** affect recipients
  - **Power, authority, and autonomy** of recipients to direct or influence the actions of themselves and/or others
  - **Presence of boundaries** between recipients
  - **General attitude** of recipients toward the innovation

**Recipient Subcodes:**

1. **Personal attributes**

Discussion of personal traits or characteristics of any recipient(s). This can include tolerance of ambiguity, general intellectual ability, motivation to change, values, goals, competence, innovativeness, seniority or tenure, learning style, being self-aware, reliable, other personality traits, etc.

*Do not use this code for recipient’s skills and knowledge.*

1. **Skills and knowledge**

Discussion about what recipients know and understand about the innovation or discussion about whether recipients have the ability/expertise to perform the tasks required for implementation.

1. **How time, resources, and support affect recipients**

Discussion about how the presence or absence of sufficient time, resources and support is affecting/affected by the ability of a specific recipient (individual or team) to implement or receive the innovation.

*For example, use this code when the interview participant says they do not have time to implement the innovation. Do not use this code when discussion is about the presence or absence of infrastructure, resources, and/or support, more generally. For example, if the interview participant states that it is always like this or staff are never given enough time to participate in QI activities. Instead, use the Characteristics of Context code, “Infrastructure, support, and resources.”*

1. **Collaboration and teamwork**

Discussion of group processes and team-related issues, including presence or absence of interprofessional collaboration, communication, and teamwork within teams, between teams and managers, and/or between individuals who work together toward a common goal; team building activities; areas of disagreement/conflict between team members or stakeholder groups; and available conflict management/resolution strategies.

*Co-code with “Networks and relationships” if discussion includes information about networks and relationships in the context in which implementation is occurring.*

1. **How existing networks affect recipients**

Discussion about how formal or informal networks and/or relationships is affecting/affected the ability and/or motivation of a specific recipient (individual or team) to implement or receive the innovation. Networks/relationships may be professional, task-related, or social and may occur at any level or across levels of the context. Examples of formal networks/relationships include memberships, listservs, communities of practice, learning communities, learning collaboratives, practice-based research networks, etc. Examples of informal networks/relationships include social practices such as getting together with colleagues for lunch; regular hallway conversations with certain colleagues; friendships; ‘huddles” among clinical providers/teams; etc.

*Use this code only when the person interviewed explicitly indicates how the network(s) and/or relationship(s) are directly affecting individual or team efforts to implement or receive the innovation.*

1. **Power, authority, and autonomy**

Discussion about the capacity or ability of an individual or team to direct or influence their own actions and/or the actions of others. Power and/or authority may be derived from organizational role (e.g., leadership), professional role (e.g., physician, nurse, etc.), expertise, relationships to powerful others, and/or ability to offer or deny rewards or use the threat of force to gain compliance.

*Co-code with the “Leadership support” (context) code when discussion is also about characteristics or behaviors of leaders that either support or hinder implementation or sustainment. When discussion includes ability of individual/teams to direct or influence others (as a characteristic of those recipients) and their use of political strategies to use/gain more power and influence, co-code with “Political factors and dynamics.”*

1. **Presence of boundaries**

Discussion/mention of recipient’s experience with boundaries between groups (e.g., professions/occupations, work units, service lines, roles) that influence implementation. Examples include discussion about the lack of communication between primary care and mental health providers and how clinicians’ scopes of practice or discipline/unit-specific restrictions limit provision of/access to services.

*If the boundaries are related to organizational structures, consider cross-coding with the “Structures and systems” context subcode. If boundaries within networks are discussed, co-code with the Context code, “Networks and relationships.”*

1. **General attitude**

Statements about how the interview participant thinks or feels about the innovation generally, e.g., that they like it or don’t like it, it is helpful, or they enjoy using it.

*Co-code with the appropriate “Innovation” subcode(s) when discussion is also about interviewee’s/recipient’s perceptions, thoughts, and/or understandings about the characteristics of the innovation.*

**Context Codes**

Two sets of Context subcodes are included in this codebook. The first set addresses the **characteristics** of context, and the second set addresses the **levels** at which these characteristics occur. At the top level, we suggest that you code only “CC. Characteristics of Context.” When subcoding text, we recommend that you first code a passage or ‘quote’ with a characteristics subcode and then co-code that text with the level of the context at which the characteristic occurred.

*Note: The i-PARIHS framework includes two characteristics of context that are not included below as subcodes: mechanisms for embedding change and environmental stability. Both of these characteristics are higher level concepts that can best be identified during later stages of the analysis process. For example, mechanisms for change may include a variety of other contextual characteristics, such as regular team meetings and performance review systems (examples of Structures and systems) or audit and feedback processes (included in Evaluation, monitoring, and feedback). Similarly, environmental stability is related to multiple contextual events/circumstances, e.g., changes or lack of changes in structures and systems, leadership, or policies and procedures. During the analysis process, we recommend that you consider whether these two higher-level characteristics have influenced implementation.*

**CC. Characteristics of Context**

Discussion about the presence or absence of the characteristics of the context in which the innovation is being introduced that may influence implementation, including:

- - **Leadership support** for innovation implementation
  - **Culture and climate** of the context
  - **History of innovation and change** in the context
  - **Policies and priorities** (as well as policy drivers and mandates) that may influence implementation
  - **Structures and systems** within the context
  - **Incentives and rewards** that encourage or discourage innovation implementation
  - **Infrastructure, resources, and support** for innovation implementation
  - **Evaluation, monitoring, and feedback** processes conducted
  - **Networks and relationships** that can influence implementation
  - **Political factors and dynamics** that may influence implementation
  - **Absorptive capacity** of the context

**Characteristics of Context Subcodes:**

1. **Leadership support**

Discussion about characteristics or behaviors of formal or informal leaders that either support or interfere with the implementation or sustainment of the innovation. This could include discussion of leadership style, relationship building, role modeling, educating, planning-organizing-aligning, and communicating, encouraging, empowering, and/or concrete support, e.g., protected time, space, resources, training, etc. Alternatively, discussion may be about how leaders fail to provide such support or exhibit negative attitudes/behaviors toward innovation implementation/sustainment.

*When discussion is about leaders providing concrete support, co-code text with “Infrastructure, resources, and support.” When discussion is also about the capacity or ability of leaders to direct or influence the actions of others, co-code with “Power, authority, and autonomy.”*

1. **Culture and climate**

Discussion about the culture of the organization or organizational unit, including prevailing norms, values, beliefs, meanings, understandings, philosophies, way of life, and assumptions. Also includes discussion about the current climate of the organization or organizational unit, e.g., staff empowerment, morale, attitudes, job satisfaction, burnout, etc., as well as the degree of stability/instability of the environment in which implementation is occurring/will occur.

*If discussion about environmental stability/instability includes reference to specific elements (e.g., leadership support variation, political dynamics in the organization), co-code with the relevant subcode (e.g., leadership support or political factors and dynamics).*

1. **History of innovation and change**

Discussion of how the organization or organizational unit has historically experienced, undertaken, and responded to past change initiatives and/or innovations.

*History of innovation and change may affect organizational absorptive capacity. Cross code with “Absorptive capacity” when informant is discussing both past experiences and current absorptive capacity, e.g., how past experiences with innovation and change have contributed to current ability to acquire, assimilate, transform, and/or apply new knowledge.*

1. **Policies and priorities**

Discussion about organizational policies, policy drivers, mandates, and/or priorities and whether/how these are related to/support/hinder the innovation and/or its implementation and the changes required. Policies are the decisions, plans, and actions that an organization, organizational unit, state, or country take to achieve specific goals. They include statements of what needs to happen and how (e.g., legislation enacted by a government, regulations or rules issued to carry out the intent of laws or of regulatory bodies, regulatory frameworks or models for enacting regulations, and organizational policies and procedures). Policy drivers are forces that influence policy decisions, e.g., serious problems, i.e., high rates of suicide; legal or ethical concerns, i.e., lack of equity; and crisis events, i.e., hurricanes and forest fires. Mandates are formal orders/commands/requirements and may be a component of written policies. Organizational priorities are identified areas of focus, e.g., improving access to care and reducing medical errors in healthcare settings.

1. **Structures and systems**

Discussion about the formal and informal ways in which the organization or organizational unit is structured and managed and/or its processes for accomplishing work. Examples of structure include authority hierarchies (e.g., chain of command), service lines, matrices, specialized or functional units or departments, inter-/multi-disciplinary teams and task forces, and decision-making levels represented in organizational charts. Although structure and systems are not always distinct, systems generally are related to organizational routines and processes, e.g., for information sharing, learning, workflow, IT, etc.

*When discussion about structures and processes is also about the ability of individuals within structures or systems to direct or influence their own actions and/or the actions of others, co-code with the Recipients code, “Power, authority, and autonomy.”*

1. **Incentives and rewards**

Discussion about mechanisms/strategies that motivate/encourage/reinforce or that deter/discourage the implementation of the innovation and proposed changes, including incentives/rewards (e.g., casual dress day; pizza day; time off; recognition; financial incentives, i.e., pay for performance; etc.) and disincentives (e.g., negative performance reviews, reprimands, regulatory requirements, etc.).

1. **Infrastructure, resources, and support**

Discussion about the presence or absence of infrastructure (e.g., facilities, space, equipment, transportation), resources (e.g., funding, staffing, time, education, skills training, materials) and/or support (e.g., supervisory, clerical) for implementing the innovation.

*Co-code with “Leadership support” when discussion attributes provision of infrastructure, resources, and support to leadership. Co-code with the Recipients “Skills and knowledge” code when discussion is also about what recipients know and understand about the innovation or whether they have the ability to perform implementation tasks. For example, when the discussion is about both knowledge/skills of staff and the lack of opportunities to learn those knowledge and skills. When discussion is also about how the presence or absence of time, resources, and/or support is affecting/affected a specific recipient’s ability to implement or receive the innovation, co-code with the Recipients “Time, resources, and support” code.*

1. **Evaluation, monitoring, and feedback**

Discussion about how the organization or organizational unit collects, assesses, monitors, and disseminates data/information about clinical processes and outcomes, economic outcomes, user experiences, clinical performance, etc. Also includes discussion about data sources (e.g., data dashboards, medical records) and ways in which results are fed back to and used by individuals, teams, and services (e.g., through presentations and/or formal reports). This information may be used, e.g., to understand current ways of working or to improve processes.

1. **Networks and relationships**

Discussion about formal or informal networks and/or relationships that may be/have been leveraged to support or hinder implementation. Networks/relationships may be professional, task-related, or social and may occur at any level or across levels of the context. Examples of formal networks/relationships include memberships, listservs, communities of practice, learning communities, learning collaboratives, practice-based research networks, etc. Examples of informal networks/relationships include social practices such as getting together with colleagues for lunch; regular hallway conversations with certain colleagues; friendships; ‘huddles” among clinical providers/teams; etc.

*Co-code with the Recipient code, “Presence of boundaries,” when discussion is also about the recipient’s experience of boundaries within or between networks that are influencing or have influenced implementation. Similarly, co-code with the Recipient code, “Collaboration and teamwork,” when discussion is also about group processes and team related issues within networks. When discussion is also about how formal or informal networks and/or relationships affected/is affecting the ability and/or motivation of a specific recipient (individual or team) to implement or receive the innovation, co-code with the Recipients code, “How existing networks affect recipients.”*

1. **Political factors and dynamics**

Discussion about organizational politics, i.e., how individuals or groups use political strategies to gain/use power and/or social influence in order to positively or negatively affect decisions and activities related to the adoption or implementation of an innovation. For example, they might create conflict, form alliances, bargain, use stalling tactics, discredit others, or compromise. If relevant, this code also includes discussion about the larger political environment (e.g., state or national government) and prevailing political ideology (e.g., nationalism, populism) as it relates to innovation implementation.

*Exclude discussion that is only about the use of sanctioned power to influence implementation, e.g., leadership support for the innovation (or lack thereof).* *When discussion about organizational politics includes discussion about the presence/absence of leadership support and/or the ability of specific individuals/teams to direct or influence the actions of others, co-code text with “Leadership support” and/or “Power, authority, and autonomy,” as appropriate. Also exclude discussion that is only about networks and relationships. When networks and relationships are being leveraged for political purposes, i.e., to gain/use power and/or social influence, co-code text with “Networks and relationships.”*

1. **Absorptive capacity**

Discussion about how the organization or organizational unit (e.g., department or clinic) identifies, acquires, assimilates, transforms, and/or applies new, valuable knowledge (e.g., evidence, Guidelines, best practices). This includes analyzing, processing, interpreting, understanding, combining with existing knowledge, and applying/incorporating new knowledge into organizational competencies and routines.

*Absorptive capacity is dynamic and influenced by the organization’s past experiences with responding to new knowledge and change. Cross-code with “History of innovation and change” if informant is discussing both current abilities and past experiences, e.g., how past experiences have contributed to current ability to acquire, assimilate, transform and/or apply new knowledge.*

**CL. Levels of Context Subcodes**

VHA Case Example of subcodes when innovation is related to a national initiative

Subcodes and definitions for the three levels of organizational context identified by i-PARIHS are provided below. However, many healthcare systems have multiple structural layers. Depending on the specific organizational context and scope of implementation effort, additional level codes may need to be applied. For example, large integrated healthcare systems, such as the U.S. Veterans Health Administration (VHA) or Kaiser Permanente, may have more than one Inner organizational context level and/or more than one Outer context level.

**CL_1. Level/Inner local context**

Local clinic where innovation is implemented

**CL_2. Level/Inner organizational context**

VA Medical Center that administratively oversees the clinic

**CL_3. Level/Regional organizational context**

VHA regional network responsible for allocating funds and meeting national standards for performance

**CL_4. Level/National organizational context**

Nationally, VHA mandates and resources that impact implementation at the local level

**CL_5. Level/Outer context**

Context outside of VHA that can impact implementation

Note: For some implementation efforts in VHA clinics that are not part of national initiatives, the regional and national levels might be considered to be part of the outer context.

The number and types of levels should be tailored to the particular study or project by the project team.

1. **Level/Inner local context**

Discussion about the immediate local setting for implementation, e.g., the clinic, office practice, hospital unit/ward/department, or nursing home.

*Co-code with Characteristics of Context subcodes.*

1. **Level/Inner organizational context**

Discussion about the larger organization within which the immediate local setting, e.g., a clinic, practice, hospital unit/ward/department, or nursing home, is embedded.

*Co-code with Characteristics of Context subcodes.*

1. **Level/Outer context**

Discussion about the broader health system, policy environment, and/or political context external to the local or inner organizational context in which implementation is taking place.

*Co-code with Characteristics of Context subcodes.*

**Facilitation Activity Codes**

This codebook focuses on **activities that facilitators perform**. However, in addition to facilitation activities, there are multiple other characteristics of Facilitation, e.g., position of facilitators in relationship to the organization (e.g., internal, external); mode of communication (e.g., virtual, in-person, telephone, email, texting); and knowledge, characteristics and skills of facilitators that can influence implementation. If for your study, you want/need to capture other characteristics of Facilitation, we encourage you to add those as subcodes to your project-specific codebook.

Three layers of codes are included for the facilitation construct: 1) the top-level code, 2) ten second level subcodes, and 3) thirty-three sub-subcodes. Facilitation is a complex implementation strategy and what facilitators do is widely variable across implementation efforts. Having two levels of subcodes will allow users of the codebook to adapt coding to project needs. The second level codes are clusters of individual activities. The sub-subcodes are individual activities and provide a finer-grained description.

**FA. Facilitation Activities**

Discussion about the activities that facilitators perform to facilitate implementation of the innovation, including:

- Providing education/information to stakeholders
- Collecting data and providing feedback to stakeholders
- Building relationships, teams, and networks to support implementation
- Enabling/fostering change in the organization
- Identifying problems and generating/selecting solutions
- Planning/preparing for implementation
- Helping to identify, define, and fill stakeholder roles
- Providing administrative and technical implementation support
- Using interpersonal skills to create a supportive environment
- Obtaining/disseminating innovation or facilitation knowledge

**Facilitation Activities Subcodes:**

1. **Providing education/information**

Educating stakeholders on clinical skills, the conduct of innovation marketing and/or organizational change processes and providing information to promote/publicize the innovation. This includes: 1) the content of education/information (e.g., information about the innovation and evidence for it, reasons for change, potential outcomes, clinical knowledge/skills needed, etc.); and/or 2) the process of providing education/information (e.g., teaching, training, mentoring, coaching, supervision, experiential/active learning, etc.).

*This is a cluster code that can be subcoded with the following activity codes: Providing education on clinical skills, Providing education on marketing, Providing education on organizational change, and Marketing.*

1. **Providing education on clinical skills**

Providing education/information on clinical skills/expertise related to the innovation. Code includes: 1) the educational content (specific clinical skills needed for the innovation); and/or 2) the educational process (e.g., teaching, training, mentoring, coaching, supervision, experiential/active learning, etc.).

*Includes making arrangements for providers to receive clinical education and if appropriate, co-code with Administrative tasks.*

1. **Providing education on marketing**

Providing education/training to stakeholders on how to conduct innovation marketing to clinic providers, staff, or leadership, including teaching innovation providers how to promote their own skills and services. Code includes: 1) the educational content (i.e., how to conduct innovation marketing, including for example how to develop a marketing plan and identify target audiences); and/or 2) the educational process (e.g., teaching, training, mentoring, coaching, supervision, experiential/active learning, etc.).

1. **Providing education on organizational change**

Providing education/information to stakeholders about organizational change processes. Code includes: 1) the educational content (e.g., information about: change skills, incentives, and resources to implement change, evaluation processes, how to engage and empower others, and quality improvement methods); and/or 2) the educational process (e.g., teaching, training, mentoring, coaching, supervision, experiential/active learning, etc.). *Exclude providing education on marketing*.

1. **Marketing**

Promoting or publicizing the innovation and its intended outcomes, including providing an evidence-base for the innovation, highlighting a need for practice change, and interpreting/translating relevant research findings for practical application.

1. **Collecting data/providing feedback**

Collecting data and other information to 1) assess and understand the local context, baseline performance, and implementation barriers/enablers; 2) collect/monitor implementation activities, progress, and outcomes; and 3) provide stakeholders with feedback on data and updates on implementation activities and relevant professional or system-level information.

*This is a cluster code which can be subcoded with the following activity codes: Conducting ongoing monitoring of innovation implementation, Data collection to assess context and baseline performance, and Providing updates and feedback.*

1. **Conducting on-going monitoring of innovation implementation**

Collecting/monitoring data or information on implementation, including progress, barriers, enablers, fidelity to evidence, provider performance, and implementation activities; also includes linking the implementation data/information to outcomes.

1. **Data collection to assess context and baseline performance**

Collecting/reviewing quantitative and/or qualitative data or information prior to active facilitation to understand the local context, baseline performance, and determinants of current practice (including barriers and enablers).

1. **Providing updates and feedback**

Providing stakeholders with information/data related to implementation processes or progress and/or interpreting the information/data. Includes providing data collected to assess context and baseline performance or to monitor implementation; and providing data/information on, for example, quality improvement activities, innovation provider activities, facilitation activities, and relevant professional or system-level information that might impact implementation.

*If updates/feedback are related to changes in the organizational context, consider co-coding with relevant context codes.*

1. **Building relationships, teams, and networks**

Engaging and building relationships with stakeholders, seeking their participation and buy-in, overcoming resistance to change, managing groups and team processes (including creating an atmosphere of mutual respect, empowering group members, and building relationships between them), and fostering stakeholder networking with peers and external experts/organizations.

*This is a cluster code that can be subcoded with the following activity codes: Engaging stakeholders, obtaining buy-in, Fostering networking with experts, Fostering peer networking, Managing group/team processes, and Overcoming resistance to change.*

1. **Engaging stakeholders, obtaining buy-in**

Engaging stakeholders who are/will be impacted by the implementation process or outcomes and seeking their participation/buy-in; including building relationships with stakeholders and/or empowering them to ‘own’ the change.

1. **Fostering networking with experts**

Helping stakeholders develop networks with external innovation or facilitation experts and/or promoting participation in such networks, e.g., linking stakeholders to experts or promoting stakeholder contact with people/organizations (non-peers) who can provide information or services.

1. **Fostering peer networking**

Helping stakeholders develop networks/relationships and communicate with similar others, within or across sites, that are, or have experience with, implementing the innovation. For example, by establishing learning collaboratives/communities of practice and/or promoting cross-pollination.

*Exclude discussion about existing peer networks and relationships and code only with the Context code, “Networks and relationships.”*

1. **Managing group/team processes**

Leading and guiding group/team processes. Examples of such activities include establishing the group; running meetings; fostering communication and teamwork; managing group dynamics; empowering group members; etc.

*Includes activities conducted as a consultant.*

1. **Overcoming resistance to change**

Working with individual leaders and providers (including innovation providers) to address/overcome resistance to implementing the innovation. Resistance may be exhibited as skepticism or negative views about the innovation or direct opposition to implementing it (e.g., by withholding information/resources/tools or attempting to negatively influence implementation processes).

*To capture specific activities facilitators used to overcome resistance, co-code with other appropriate activity codes, e.g., “Engaging stakeholders/obtaining buy-in,” “Marketing,” and/or the various education codes.*

1. **Enabling/fostering change**

Encouraging, promoting, and helping to support changes in the organization, including by interceding and liaising with leadership or other stakeholders and assisting with the development of strategies and policies. The target of change efforts may be the organizational structure or culture or the target of change may not be specified but the methods of fostering change are specified. (For example, discussion may be about assisting stakeholders with conducting quality improvement activities, helping them build capacity for sustainment, or guiding and supporting them during the implementation process.)

*This is a cluster code that can be subcoded with the following activity codes: Fostering organizational change: cultural, Fostering organizational change: structural, Fostering change/unspecified, Interceding and liaising with others, and Assisting with strategy/policy development.*

1. **Fostering organizational change: culture and climate**

Promoting changes in the shared norms, values, beliefs, meanings, understandings, philosophies, and assumptions of the organization or one of its sub-groups (e.g., primary care or mental health providers); and/or promoting changes in climate such as empowering staff, or improving morale, attitudes, job satisfaction, etc. Examples of activities target culture include promoting changes in shared beliefs about care, providers’ skills and scopes of practice, efficacy and safety of treatments and practice models; and promoting changes in norms related to openness to change. Examples of activities targeting climate include advocating for actions that might improve morale, and/or making recommendations to assess job satisfaction and reduce burnout. *This code may overlap with the context code “Culture and climate.” Co-code with the “Culture and climate” context code when discussion provides information about the culture or climate of the context in addition to the specific activities facilitators do to promote changes to existing culture or climate.*

1. **Fostering organizational change: structural**

Promoting changes in the factors that affect: 1) the ways in which the organization or unit is structured and managed, 2) its processes for accomplishing work, and/or 3) the physical context. Examples include changes in organizational structure, staff roles, referral methods, workflows, and space/office assignments. *This code may overlap with the context codes “Structures and systems” and/or “Infrastructure, resources, and support.” Co-code with the appropriate context code when discussion provides information about these characteristics in addition to the specific activities facilitators do to promote changes to existing structures, systems, or infrastructure. Exclude discussion about activities performed to actively assist sites in hiring clinical staff and use the Facilitation Activities code, “Helping to hire clinical program staff.”*

1. **Fostering change, unspecified**

Promoting change generally to support implementation, i.e., the target of change is not specified. Discussion may, however, include the methods used for promoting change, e.g., facilitator assisted stakeholders with conducting Quality Improvement (QI) activities such as Plan-Do-Study-Act (PDSA) cycles.

1. **Interceding and liaising with others**

Interceding/liaising directly with leadership and/or other relevant stakeholders/programs/departments (at any level) about stakeholder needs and/or the innovation (including regulatory issues, facilitation implementation, other issues).

1. **Assisting with strategy/policy development**

Helping stakeholders, particularly leaders, develop/revise organizational strategies and/or policies to support innovation implementation. This may be done within the organization (e.g., helping to revise a facility-wide suicide prevention policy, or participating in strategic planning processes to ensure innovation infrastructure support) or outside of the organizational entity (e.g., by informing policies that may impact innovation implementation).

1. **Problem identification and resolution**

Conducting or helping stakeholders 1) identify, become aware of, or clarify implementation challenges/barriers/problems and/or 2) generate potential solutions/countermeasures or select the one(s) most likely to address/solve implementation challenges/barriers/problems.

*This is a cluster code and can be subcoded with the following activity codes: Problem identification, Problem solving.*

1. **Problem identification**

Assisting stakeholders with identifying, becoming aware of, and/or clarifying implementation challenges/barriers/problems. Includes, for example, understanding the current ways of working and thinking and identifying contextual gaps and barriers to implementation.

*Activity may be conducted by facilitator alone or by facilitator working with site stakeholders. Challenges/problems/barriers may be related to the context, the innovation and/or the recipients; co-code with subcodes for those constructs, if appropriate.*

1. **Problem-solving**

Assisting stakeholders with generating potential solutions/countermeasures and/or selecting the one(s) most likely to address/solve implementation challenges/barriers/problems.

*Activity may be conducted by facilitator alone or by facilitator working with site stakeholders. Challenges/problems/barriers may be related to the context, the innovation and/or the recipients; co-code with subcodes for those constructs, if appropriate.*

1. **Planning/preparing for implementation**

Helping stakeholders develop or refine Action/Implementation plans, come to consensus, adapt the innovation to the local context (structure, staffing, culture, and other initiatives), share a vision for change, and identify goals and priorities.

*This is a cluster code that can be subcoded with the following activity codes: Action/implementation planning, Adapting innovation to local context, Developing shared vision/consensus building, and Setting goals/priorities.*

1. **Action/implementation planning**

Assisting with the development and refinement of tasks and strategies for implementing the innovation, including deciding formal steps that need to be taken, short or long-term plans; also assisting sites in completing a formal ‘Action’ or ‘Implementation’ Plan/Checklist.

1. **Adapting innovation to local context**

Helping to tailor the innovation to the local context (including local structure, staffing, and culture) and/or create synergy with other initiatives without compromising fidelity.

1. **Developing shared vision/consensus building**

Finding synergy between existing goals and the innovation and/or implementation goals; helping stakeholders to “get on the same page” or to see what is in it for “me and us;” and developing “win-win solutions.”

1. **Setting goals/priorities**

Assisting in setting or reviewing and revising clear, realistic goals and priorities to support implementation. Includes assisting with the selection of an area for change and developing/refining specific clinical practice questions.

1. **Helping to define, identify and fill stakeholder roles**

Helping to identify and select local change agents (e.g., facilitators, QI team members, local champions, opinion leaders) and/or hire innovation providers, as well as establish, describe/clarify, and/or allocate facilitator and stakeholder roles and responsibilities.

*This is a cluster code and can be subcoded with the following activity codes: Describing/clarifying roles and responsibilities, Helping to hire clinical program staff, Helping identify/select local change agents.*

1. **Describing/clarifying roles and responsibilities**

Describing the purpose and process of the clinical innovation, facilitation, and the activities that will occur; and establishing, clarifying, and/or allocating the roles and responsibilities of individuals involved in implementation processes.

1. **Helping to hire clinical program staff**

Assisting with hiring innovation providers, replacements, or additions, including helping write job/position descriptions.

*Exclude discussion about promoting the need to hire staff and code with “Fostering organizational change: structural.”*

1. **Helping Identify/select local change agents**

Helping identify and/or select local change agents, e.g., internal facilitators, opinion leaders, champions, and quality improvement team members.

*Exclude clinical providers and code with “Helping to hire clinical program staff.”*

1. **Providing administrative/technical support**

Conducting administrative tasks that support the operationalization of implementation activities and providing technical support, i.e., practical help and assistance to support implementation. Examples of administrative tasks include arranging calls, meetings, and implementation site visits; developing/preparing and disseminating minutes/reports and educational/marketing materials; and organizing innovation provider training. Examples of technical support include providing tools/sample materials; working with site stakeholders to co-create tools/materials, identifying/providing information about available resources for implementation, and working with relevant stakeholders to ensure that Information Technology (IT) systems accurately capture innovation activity and support implementation.

*This is a cluster code and may be subcoded with the following activity codes: Administrative tasks, Technical support*.

1. **Administrative tasks**

Conducting administrative tasks that support the operationalization of implementation activities. Examples include arranging calls, meetings, and implementation site visits; developing/preparing and disseminating minutes/reports and educational/marketing materials; and organizing innovation provider training.

*When administrative tasks overlap with other facilitation activities, co-code for those activities. For example, if the facilitator is arranging to send an innovation provider to another clinic to shadow innovation providers there, co-code with “Administrative tasks” and “Providing education on clinical skills.”*

1. **Technical support**

Providing practical help and assistance to support implementation activities. Examples of such activities include providing tools/sample materials; working with site stakeholders to co-create tools/materials, identifying/providing information about available resources for implementation, working with relevant stakeholders to ensure that Information Technology (IT) systems accurately capture innovation activity and support implementation (e.g., by providing support for computer/software issues), or reviewing, appraising, and/or summarizing the evidence for the innovation.

1. **Using interpersonal skills to create a supportive environment**

Using positive, supportive behaviors and communications to create an open, supportive, and trusting environment conducive to change, including being generally helpful and available, communicating regularly, acknowledging ideas and efforts, and celebrating achievements/success. This code also includes selectively reducing the level of facilitation support, including positive supportive behaviors, in order to allow the transfer of facilitation roles to site stakeholders.

*This is a cluster code that can be subcoded with the following activity codes: Providing support using interpersonal skills, Pulling back/transferring roles.*

1. **Providing support using interpersonal skills**

Using positive, supportive behaviors and communications to create an open, supportive, and trusting environment conducive to change. Examples include being generally helpful and available, communicating regularly, maintaining enthusiasm, providing encouragement and exhibiting empathy, acknowledging ideas and efforts, and celebrating achievements/success. May also include self-disclosure (sharing personal insights or experiences) or interjecting humor.

*If facilitator does not exhibit positive supportive behaviors or exhibits negative behaviors (e.g., being hypercritical), co-code with “Barriers.”*

1. **Pulling back/transferring roles**

Becoming less active (i.e., reducing facilitation support) and decreasing involvement/presence to allow and encourage site stakeholders to assume responsibility for supporting implementation/sustainment. For example, when problems arise, rather than jumping in immediately, waiting to see if site stakeholders can problem solve and identify solutions without input.

1. **Obtaining/disseminating innovation or facilitation knowledge**

Obtaining information about/developing skills needed for facilitating implementation of the innovation or fostering dissemination of knowledge about the innovation or facilitation other than at the implementation site(s). Facilitators may foster dissemination by attending, presenting at, or organizing non-local meetings or by assisting with dissemination at sites not receiving facilitation.

*This is a cluster code that can be subcoded with the following activity codes: Attending, presenting at, and/or organizing non-local meetings; Fostering spread of clinical innovation/facilitation methods; Obtaining training/continuing education.*

1. **Attending, presenting at, and/or organizing non-local meetings**

Organizing, attending and/or presenting on the innovation and the implementation effort at regional/national meetings, councils, advisory boards, or other forums.

*Exclude visits to participating sites*.

1. **Fostering spread of clinical innovation/facilitation methods**

Assisting with dissemination of the clinical innovation and/or facilitation methods at sites not receiving implementation facilitation.

1. **Obtaining training/continuing education**

Obtaining information about and/or developing skills needed for facilitating implementation of the innovation. Examples of training/education content include facilitation activities/processes, innovation details/evidence, organizational change processes, and resources available to support change. Education/training may be obtained by attending trainings, meetings, webinars, reading, or obtaining consultation from experts in facilitation or the innovation.
